# Dissecting the multi-omics atlas of the exosomes released by human lung adenocarcinoma stem-like cells

**DOI:** 10.1038/s41525-021-00217-5

**Published:** 2021-06-14

**Authors:** Hai-Tao Luo, Yuan-Yuan Zheng, Jun Tang, Li-Juan Shao, Yi-Heng Mao, Wei Yang, Xiao-Fei Yang, Yang Li, Rui-Jun Tian, Fu-Rong Li

**Affiliations:** 1 Translational Medicine Collaborative Innovation Center, Shenzhen People’s Hospital (The Second Clinical Medical College, Jinan University; The First Affiliated Hospital, Southern University of Science and Technology), Shenzhen, Guangdong China; 2 Shenzhen Key Laboratory of Stem Cell Research and Clinical Transformation, Shenzhen, China; 3 Integrated Chinese and Western Medicine Postdoctoral Research Station, Jinan University, Guangzhou, China; 4 Institute of Oncology, Shenzhen People’s Hospital (The Second Clinical Medical College, Jinan University; The First Affiliated Hospital, Southern University of Science and Technology), Shenzhen, Guangdong China; 5 Department of Chemistry, Southern University of Science and Technology, Shenzhen, China

**Keywords:** Computational biology and bioinformatics, Cancer stem cells

## Abstract

Lung adenocarcinoma is heterogeneous and hierarchically organized, with a subpopulation of stem-like cells (CSCs) that reside at the apex of the hierarchy, in which exosomes act as important mediators by transporting specific molecules among different cell populations. Although there have been numerous studies on tumor exosomes, the constituents and functional properties of CSC-derived exosomes are still poorly characterized. Here we present a detail transcriptome and proteome atlas of the exosomes released by human lung adenocarcinoma stem-like cells (LSLCs). The transcriptome analysis indicates the specific patterns of exosomal constituents, including the fragmentation of transcripts and the low-level presence of circular RNAs, and identifies multiple exosomal-enriched mRNAs and lncRNAs. Integrative analysis of transcriptome and proteome data reveals the diverse functions of exosomal-enriched RNAs and proteins, many of which are associated with tumorigenesis. Importantly, several LSLC markers we identified are highly expressed in LSLC-derived exosomes and associate with poor survival, which may serve as promising liquid biopsy biomarkers for lung adenocarcinoma diagnosis. Our study provides a resource for the future elucidation of the functions of tumor-derived exosomes and their regulatory mechanisms in mediating lung cancer development.

## Introduction

A solid tumor has been viewed as a community^1–3^, in which cells communicate with each other and are hierarchically organized into distinct functional populations^4,5^, such as cancer stem cells (CSCs) that are defined as “cancer root cells” and exhibit self-renewal and repopulation capacity^6–8^. Intercellular communications via molecular transfer, either between CSCs and resident tumor cells or between tumor cells and their microenvironments, are key determinants of cancer development and metastasis^9–11^. Currently, several intercellular communicators have been identified such as microvesicles, exosomes, and non-vesicular carriers^12–14^. Exosomes are endosome-derived nanovesicles (40–150 nm) secreted by nearly all cell types and released into the extracellular space^15^. As important mediators, exosomes secreted by cancer cells contain both coding and non-coding RNAs, as well as proteins, which can influence the formation and homeostasis of a tumor niche and microenvironment^12,16^. Furthermore, circulating exosomes released by CSCs may carry cancer recurrence- or therapy resistance-associated markers, which may act as therapeutic targets for CSCs or serve as promising liquid biopsy biomarkers for cancer diagnosis^17–19^. Nevertheless, the detail characterization and functional interpretation of exosomes secreted by CSCs at both the transcriptomic and proteomic levels have not been sufficiently addressed to date.

In this work, we focused on human lung adenocarcinoma, the most common type of lung cancer, as a research model for systematic investigation of intrinsic composition and functional properties of cancer-released exosomes. Currently, the specific markers of lung adenocarcinoma stem cells are still debated in the field. According to previous studies, aldehyde dehydrogenase (ALDH) has been identified as a CSC marker^20^ and extensively used for isolating CSCs from several cancer types, including breast cancer, ovarian cancer, and melanoma^21–23^. We found that ALDEFLUOR-positive subpopulations from lung adenocarcinoma cell lines display strong CSC-like properties and thus are viewed as lung adenocarcinoma stem-like cells (LSLCs). Based on multi-step ultracentrifugation, exosomes from both LSLCs and lung adenocarcinoma bulk cells (LBCs) were obtained and examined by transmission electron microscopy (TEM) and nanoparticle tracking analysis (NTA). Moreover, parallel analysis of LSLCs and their exosomal (LSLC-EXO) transcriptomes generated by Ribo-zero RNA sequencing (RNA-seq) technology, along with LBCs and their exosomes (LBC-EXO), enabled us to depict a detail map of exosomal RNAs, including mRNAs, long non-coding RNAs (lncRNAs) and circular RNAs (circRNAs).

Proteome profiling of CSC-derived exosomes is uniquely challenging, largely due to their low abundance. Recently, we developed the simple and integrated spin-tip-based proteomics technology (termed SISPROT) that enabled seamless integration of multiple steps of proteomics sample preparation, desalting, and high-pH reversed phase fractionation into a single spin-tip device^24^. The full integration design of the SISPROT technology has been approved to significantly increase the proteome profiling sensitivity and throughput^25–27^. Benefiting from the high sensitivity of the SISPROT technology, thousands of proteins were identified from LSLCs and their exosomes. The combined analysis of transcriptome and proteome data shows great promise in characterizing the exosomal-enriched components as well as LSLC-specific RNAs or proteins. Many exosomal biogenesis-related or cancer-related pathways, such as extracellular matrix (ECM) organization, regulation of ion transmembrane transport, cell adhesion, and cell migration, are enriched with exosomal- or LSLC-specific genes. We demonstrated that several RNAs and proteins that are specifically expressed in LSLCs and associated with poor survival in lung cancer patients are packaged into exosomes, which present a source of liquid biopsy biomarkers in lung adenocarcinoma diagnosis and prognosis.

## Results

### ALDEFLUOR-positive lung adenocarcinoma cells display CSC-like properties

In order to evaluate the presence and the proportion of CSCs, ALDH enzymatic activity was assessed using the ALDEFLUOR assay in three lung adenocarcinoma cell lines with different *KRAS* and *EGFR* mutational status: two *KRAS* mutant cell lines (A549 and H1734) and one *EGFR* mutant cell line H1975. An average of 2% ALDEFLUOR-positive cells was observed in three cell lines (Supplementary Fig. 1a). Notably, the proportion of ALDEFLUOR-positive cells in H1975 (3.5%) was significantly higher than that in A549 (1.33%) and H1734 (1.27%) (Supplementary Fig. 1b). Interestingly, the CD44/CD24 ratio, which positively correlates with the malignance of cancer, was the highest in H1975 (Supplementary Fig. 2a). These results suggested that 1.27–3.5% of cells from three cell lines have stemness potential, with H1975 having the largest proportion.

**Fig. 1:**
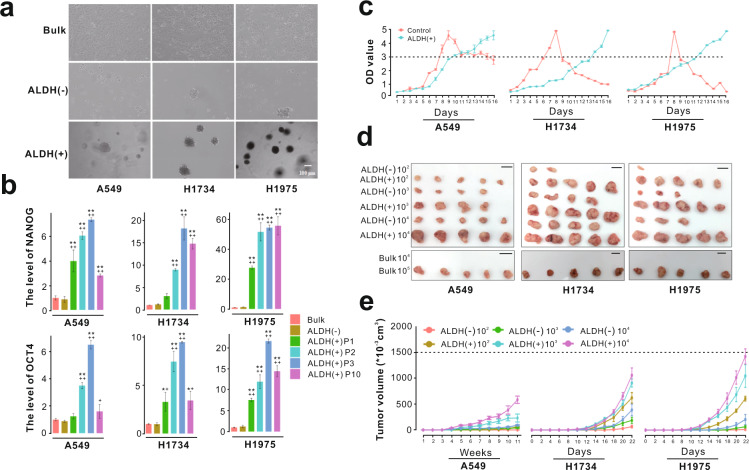
The ALDEFLUOR-positive cells from human lung adenocarcinoma have cancer stem-like cell properties. **a** The bulk cells, ALDEFLUOR-negative cells, and ALDEFLUOR-positive cells from three lung adenocarcinoma cell lines were cultured with serum-free suspension 3D culture system for 7 days. **b** The levels of stem genes (NANOG and OCT4) of bulk cells, ALDEFLUOR-negative cells, and ALDEFLUOR-positive cells with different passage from three cell lines were analyzed by qPCR. **p < 0.01, *p < 0.05, when compared with bulk cells. ^++^*p* < 0.01, ^+^*p* < 0.05, when compared with ALDEFLUOR-negative cells. **c** The cell growth rates were determined by CCK-8 assay. The bulk cell population was used as control. **d** In vivo mice tumor development was evaluated after subcutaneous injection of different number of ALDEFLUOR-negative and ALDEFLUOR-positive cells. ALDEFLUOR-positive cells were injected to the right side of mice while the same number of ALDEFLUOR-negative cells were injected to the left side of the same mice. Experiments were performed in quintuplicate. Scale bar, 1 cm. **e** The volume of solid subcutaneous tumor changed over time (error bars represent ±SEM).

ALDEFLUOR-positive and ALDEFLUOR-negative cells were sorted by flow cytometry for each cell line to detect their CSC-like phenotypes, including sphere formation, clonogenicity, stem cell marker expression, proliferative capacity, and tumorigenicity. First, the tumor sphere formation assay was used to measure the self-renewal capacity. The ALDEFLUOR-positive cells sorted from three cell lines were capable of forming spheres after culture for 7 days in a serum-free suspension three-dimensional (3D) culture system, whereas the ALDEFLUOR-negative cells and bulk cells displayed weak sphere formation capacity (Fig. 1a). After culture for 21 days, ALDEFLUOR-positive cells from H1975 formed the largest tumor spheres compared with those from A549 and H1734 (Supplementary Fig. 2b, c). The results from colony formation assay demonstrated that clonogenicity was remarkably enhanced in ALDEFLUOR-positive cells (Supplementary Fig. 2d, e). Moreover, the well-known stem cell markers including NANOG and OCT4 were highly expressed in multiple passages of ALDEFLUOR-positive cells with passage 3 showing the highest and most stable level of the two stem genes (Fig. 1b and Supplementary Fig. 2f). Thus, the ALDEFLUOR-positive cell spheroids of passage 3 were selected as the candidate CSC model in subsequent experiments. In addition, in comparison with the bulk cell population, the cell proliferative capacity increased progressively over time in the ALDEFLUOR-positive population according to the results of Cell Counting Kit-8 assay (Fig. 1c). Finally, the tumorigenicity of ALDEFLUOR-positive cells was evaluated in animal models. ALDEFLUOR-positive cells were injected subcutaneously in the right side of BALB/c nude mice while the same numbers of ALDEFLUOR-negative cells were injected in the left side of the same mice. Experiments were performed in quintuplicate. The results showed that, when 1 × 10^2^ cells were injected, all ALDEFLUOR-positive cells generated tumors, while ALDEFLUOR-negative cells failed to reproducibly form tumors (Fig. 1d). The bulk cells also failed to form tumor, even when 1 × 10^4^ cells were injected. Meanwhile, the size of tumor formation was positively associated with the number of ALDEFLUOR-positive cells injected (Fig. 1e). The terminal tumor weights generated by ALDEFLUOR-positive cells were significantly higher than those generated by ALDEFLUOR-negative cells (Supplementary Fig. 3). On the other hand, the tumor volumes increased sharply in the ALDEFLUOR-positive groups compared with the ALDEFLUOR-negative groups (Fig. 1e). Hematoxylin and eosin (H&E) staining of xenograft tumor sections confirmed the ubiquitous presence of malignant cells in tumors formed by ALDEFLUOR-positive cells (Supplementary Fig. 4a). We also confirmed the high expression of ALDH1 in tumors formed by ALDEFLUOR-positive cells by immunostaining using the ALDH1 antibody (Supplementary Fig. 4b).

Taken altogether, the above results indicated that ALDEFLUOR-positive lung adenocarcinoma cells display CSC-like properties including self-renewal, strong proliferative capacity, and tumorigenicity, therefore we consider ALDEFLUOR-positive cells as LSLCs. The results also showed that ALDEFLUOR-positive cells from H1975 exhibited stronger CSC characteristics than those from A549 and H1734, thus ALDEFLUOR-positive cells from H1975 were selected and used as LSLC model for subsequent analysis.

### Isolation and characterization of LSLC-EXO and LBC-EXO

**Fig. 2:**
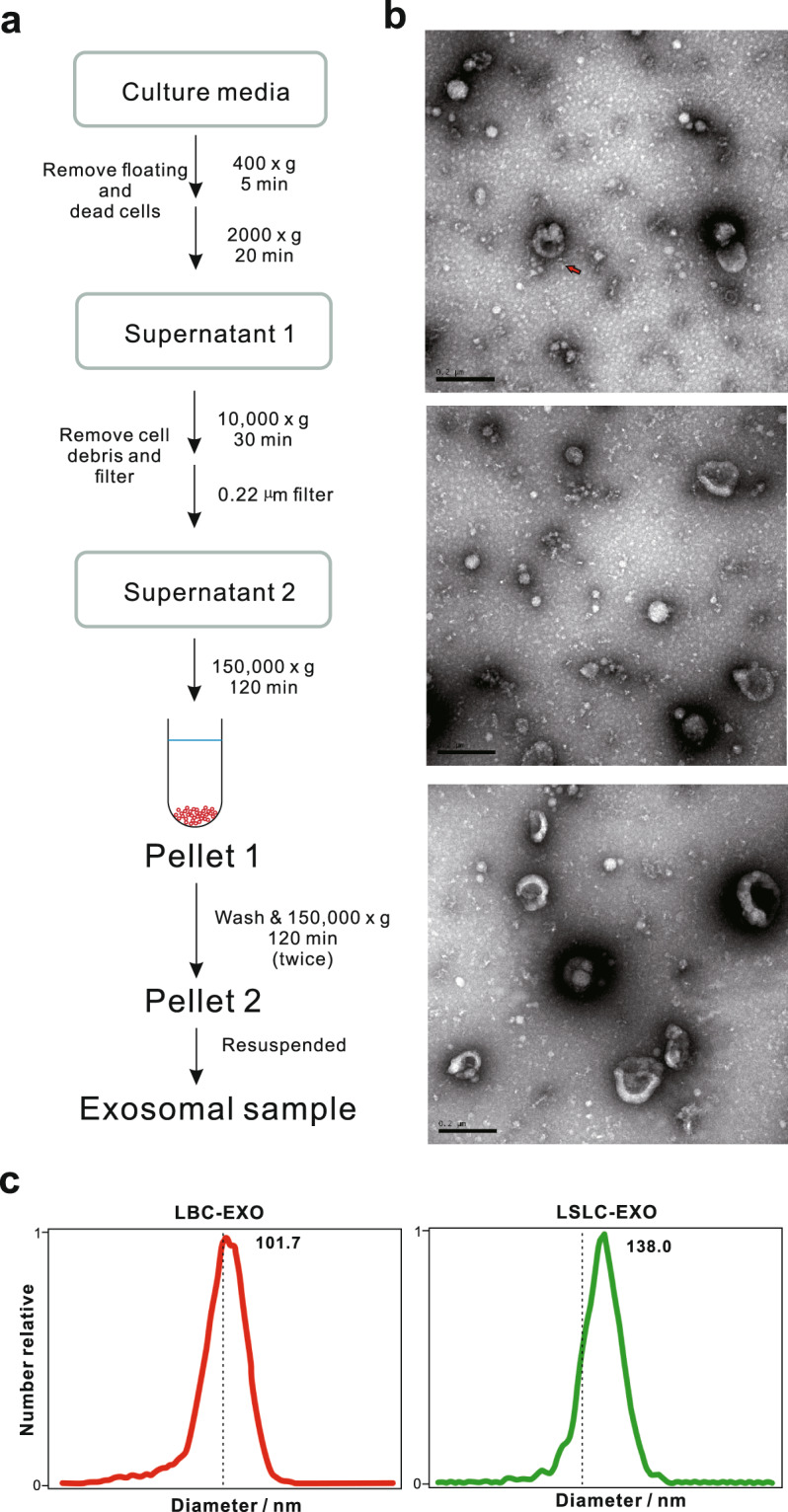
Isolation and biophysical properties of exosomes. **a** Schema for isolation of exosomes using multi-step ultracentrifugation. **b** Transmission electron microscopy (TEM) imaging of exosomes isolated from three replicates of LSLC. Scale bar, 200 nm. **c** Size distribution profiles of exosomes by nanoparticle tracking analysis (NTA).

To study the constituents and functional properties of LSLC-EXO, exosomes released by LSLC as well as LBC that was used as control, were isolated by multi-step ultracentrifugation^28,29^ (Fig. 2a). The presence of isolated exosomes exhibiting cup-shaped morphology with a diameter size range of 40–150 nm was confirmed by TEM (Fig. 2b and Supplementary Fig. 5a). In addition, compared with the whole-cell lysates of bulk cells, the classical exosomal markers CD9, CD63, and CD81 were highly expressed in exosomes (Supplementary Fig. 5b). Then the size distribution of exosomes was evaluated by NAT that has been widely applied for sizing the particles in liquids. According to NAT, the size of exosomes from LBCs and LSLCs were about 101.7 and 138.0 nm, respectively (Fig. 2c). These results indicated that exosomes derived from LSLCs and LBCs were isolated successfully.

### LncRNAs and intronic fragments are enriched in exosomes

**Fig. 3:**
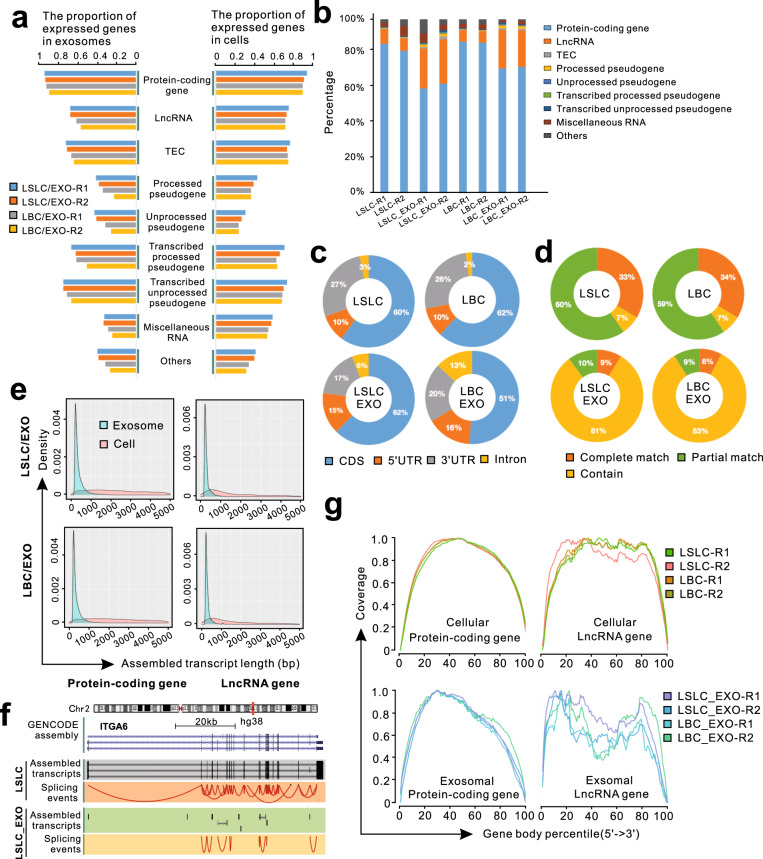
Composition analysis of diverse RNA types in exosomes and their parental cells. **a** The proportion of each gene type identified in exosomes (left panel) and cells (right panel) at the transcriptomic level. **b** Normalized reads distribution of each gene type for each sample. **c** Percentage of RNA-seq reads mapping to exonic (CDS, 3’UTR, and 5’UTR for protein-coding gene) and intronic gene regions for cellular and exosomal samples. **d** The statistics of assembled transcripts that matched to reference protein-coding genes. **e** The length distribution of reference-matched transcripts assembled from exosomes and cells. **f** The genomic view and splicing events of *ITGA6* gene. **g** The read coverage over gene body.

In order to characterize the repertoire of exosomal RNAs, we performed Ribo-zero RNA-seq, which can capture both polyA and non-polyA RNAs, on LSLCs and LBCs as well as their derived exosomes, with two replicates for each. Specifically, total RNAs from each sample were isolated and ribosomal RNAs (rRNAs) were then removed by the Epicentre Ribo-zero™ rRNA Removal Kit. The remaining long polyA and non-polyA RNAs were sequenced by Illumina Hiseq 4000 platform with 150 bp paired-end reads. The clean reads from each sample were mapped to reference gene models (GENCODE database v35) using the pseudoalignment-based tool Kallisto^30^. On average, we detected 39,928 and 38,043 intracellular genes with the read counts of more than one in LSLC and LBC, whereas 37,871 and 33,913 genes were detected in LSLC-EXO and LBC-EXO, respectively. Specifically, an average of 89.5% protein-coding genes and 71.7% lncRNAs annotated by GENCODE database^31^ were expressed (the read counts of more than one) in cellular samples (Fig. 3a). The similar proportion of genes was expressed in exosomes (90.5% for protein-coding genes and 62.2% for lncRNAs). The RNA fractions of LSLCs and LBCs exhibited common characteristics (Fig. 3b). Although the protein-coding gene was the most abundant across all samples, the lncRNA fractions were larger for exosomal samples compared with their parental cells (Fig. 3c). Furthermore, for both protein-coding and lncRNA genes, the reads from intronic regions were more enriched in exosomes than in bulk cells (increased by 2–6.5 times; Fig. 3c and Supplementary Fig. 5c). The enrichments of lncRNAs and intronic reads that had been validated by previous studies^14,28^ suggested that intracellular RNAs and intronic sequences may be selectively packaged into exosomes.

### Exosomal RNAs are fragmented and tend to come from 3′- or 5′-ends

To further investigate the intactness of the exosomal RNAs, we performed de novo transcriptome assembly for each sample without the aid of human reference genome using the StringTie method^32^. First, the clean reads were aligned to the human reference genome using STAR aligner^33^, then the transcripts were assembled by StringTie. The assembly of each sample was compared with reference protein-coding and lncRNA gene models^31^. The assembled transcripts matching to the reference gene models were classified into three categories, including complete matched transcripts whose intron chains were identical with reference models, partially matched transcripts when at least one splice junction was identical with reference models, and contained transcripts when they were contained in reference models. Strikingly, we found that the overwhelming majority (around 90%) of exosomal RNAs belonged to contained transcripts, whereas the percentage was only 6–7% for cellular RNAs (Fig. 3d and Supplementary Fig. 5d). The length distributions of assembled transcripts exhibited the distinct patterns between exosomal and cellular RNAs, with an average length of only 359 nucleotides for exosomal RNAs (Fig. 3e). These results demonstrated that the transcripts involved in exosomes were fragmented, which was further reflected by the lower number of splices presented in exosomes (an average of 5,982,926) compared with parental cells (an average of 40,698,750) (Supplementary Fig. 5e). For example, the assembled transcripts of *ITGA6* from LSLC samples, which has been reported as a marker of CSCs^34^, were complete matched with the reference model, suggesting that the intact *ITGA6* mRNA transcripts are present in LSLCs. In comparison, only a few short fragments of *ITGA6* were observed in LSLC-EXO, suggesting that the *ITGA6* transcripts presented in exosomes were fragmented (Fig. 3f). Moreover, according to the distribution of read coverage across the entire gene bodies, we found that the exosomal fragments tended to come from the regions of 3′- or 5′-ends of lncRNAs with only a slight tendency to come from 3′-ends of mRNAs (Fig. 3g).

### The distinct signatures of RNA variants between cellular and exosomal RNAs

**Fig. 4:**
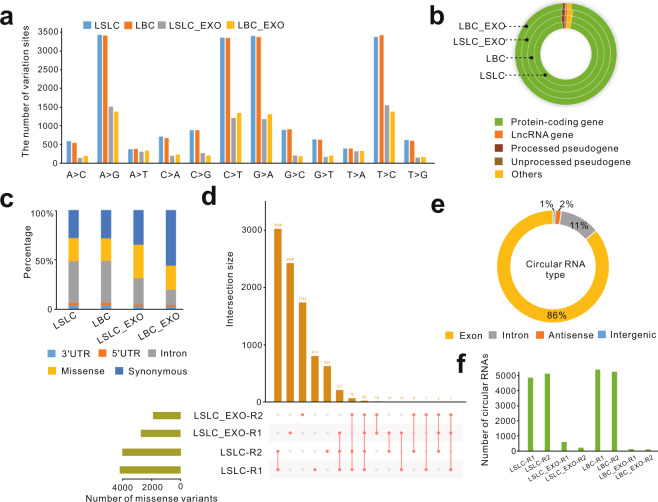
The statistics of RNA variants and circular RNAs. **a** Distribution of variant types identified from cellular and exosomal transcriptomes. **b** Distribution of RNA variants in different gene types. **c** Distribution of RNA variants in different regions of protein-coding genes. **d** Matrix layout for all intersections of LSLC and LSLC-EXO samples, sorted by intersection size. Pink dots in the matrix indicate sets that are part of the intersection. **e** The proportion of circRNAs from different genomic regions. **f** The number of circRNAs identified from LSLC and LSLC-EXO samples.

LSLC-specific mutation signatures that are carried by LSLC-EXO may serve as clinical liquid biopsy biomarkers for cancer diagnostics and prognostics^35^. Although numerous studies showed that cancer specific mutations could be detected in circulating tumor DNA^36–38^, few studies have explored the signatures of exosomal genetic variants in cancer. To examine whether genetic variants in cancer cells at RNA level were also present in their derived exosomes, we performed variant calling analysis by Genome Analysis Tool Kit (GATK) using our Ribo-zero RNA-seq data. The mean of the number of single-nucleotide variants (SNVs) was 18,571 and 7309 for cellular and exosomal transcriptomes, respectively. The most common changes observed were substitutions between A/T and G/C (Fig. 4a and Supplementary Fig. 6a). An average of 96% of variants were located in protein-coding genes (Fig. 4b); 26% were missense substitution variants (Fig. 4c). Notably, we found an average 82% (3348/4094) of missense variants were shared between two replicates of LSLCs, whereas only a very small number of missense variants were shared either between two replicates of LSLC-derived exosomes (2%) or between LSLCs and their derived exosomes (an average 6%) (Fig. 4d and Supplementary Data 1). The results of LBCs obtained were of similar patterns (Supplementary Fig. 6b). These observations suggested that utilizing RNA variant profiles of cancer cell-derived exosomes may not reflect the events occurring in cancer cells.

### Few circRNAs presented in exosomes

As a novel type of RNA, circRNAs that are generated by pre-mRNAs through back-splicing processes are highly stable and could be used as potential biomarkers of cancer^39,40^. Based on Ribo-zero RNA-seq data, we can reliably identify back-spliced events, which have been viewed as the gold standard methods for identification and quantification of circRNAs^39,41^. In this way, we fully identified 21,365 candidate circRNAs using CIRIquant algorithm that utilized the pseudo-reference alignment strategy and exhibited high performance in accurate circRNA identification and quantification^42^. To further validate the reliability of our identified circRNAs, we compared them with human reference circRNAs annotated by circAltas 2.0 database^43^. The result showed that 71.7% (15,318/21,365) of circRNAs were matched with reference models and used in the following analysis (Supplementary Data 2). Furthermore, consistent with previous findings^39^, we found that 86% of circRNAs were generated from exonic regions and were supported by the back-spliced exon–exon junctions (Fig. 4e). Of note, compared with the large amount of circRNAs involved in LSLCs and LBCs (an average of 5125), there were only an average of 274 presented in their derived exosomes (Fig. 4f). We inferred that the few circRNAs detected in exosomes may due to the fragmentation of exosomal RNAs. Nevertheless, the roles of these exosomal-enriched circRNAs, especially for those from LSLC-EXO, need further investigation.

### Cancer-derived exosomes more closely reflect cancer cells at the proteomic level

**Fig. 5:**
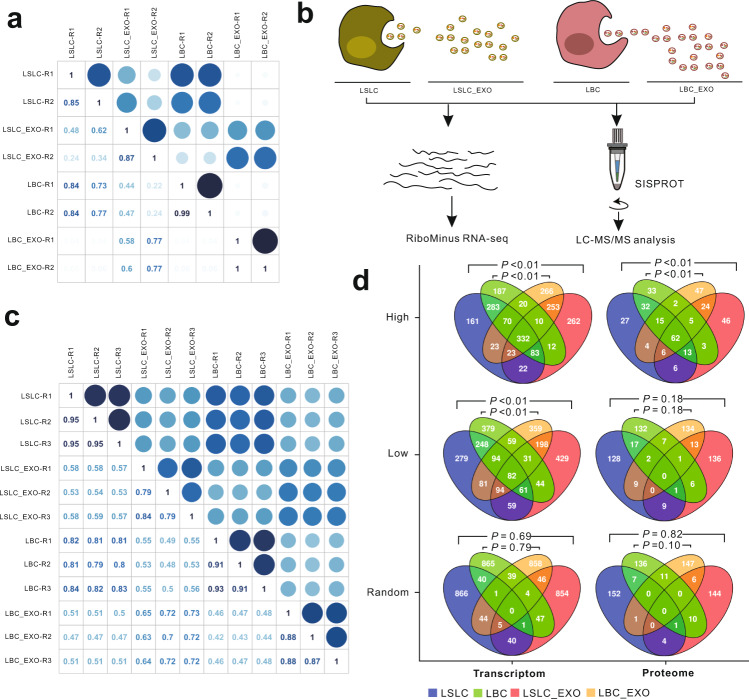
The correlation analysis of cellular and exosomal RNAs and proteins. **a** The protein-coding gene expression correlation matrix based on transcriptomic data of all samples. Color intensity and the size of the circle are proportional to the Pearson correlation coefficients. **b** Scheme of the transcriptomic and proteomic data generation. **c** The gene expression correlation matrix based on proteomic data of all samples. **d** Venn diagrams depict the overlap of protein-coding genes (left panel) and proteins (right panel) among LSLC, LSLC-EXO, LBC, and LBC-EXO samples. *P* values were calculated by hypergeometric tests.

To further investigate the degree of resemblance between cancer cells and their exosomes, we conducted correlation analysis by the combination of transcriptomic and proteomic data. First, expression profiles of both protein-coding and lncRNA genes of two replicates in each sample exhibited high correlations (the mean of Pearson correlation coefficients was 0.93) (Fig. 5a), suggesting the good repeatability and minimal batch effects of our experiments. The gene expression correlation between LSLCs and LBCs was relatively higher than those between their derived exosomes. Interestingly, we found the expression correlation of lncRNA genes between LSLC-EXO and LBC-EXO (average 0.87) was higher than those of protein-coding genes (average 0.68) (Supplementary Fig. 7a). Furthermore, very weak gene expression correlations were observed either between LSLC and LSLC-EXO (average 0.22) or between LBC and LBC-EXO (average 0.10).

Next, to examine whether exosomes can more closely mirror their parental cells at the proteomic level, we performed proteome profiling experiments (Fig. 5b). Specifically, an integrated method termed SISPROT, which has been proved as an efficient and reproducible way for proteomic sample preparation especially for limited amount of cell samples^25–27^, was used to extract and digest proteins from LSLCs and LBCs as well as their exosomes. Tryptic peptides were analyzed by liquid chromatography tandem mass spectrometry (LC-MS/MS) based on a Q-Exactive HF-X MS. Raw proteomic data were searched against the human Uniprot database using MaxQuant^44^ and a label-free quantitative method was applied to quantify differentially expressed proteins. For each sample, experiments were performed in triplicate. According to the results of the proteomic profiling analysis, an average of 2676 and 1017 proteins were identified in cellular and exosomal samples, respectively (Supplementary Data 3). The high correlations of protein expression among three replicates for each sample (within the 0.79–0.95 range) indicated the robustness and good reproducibility of our sample preparation and LC-MS/MS experiments (Fig. 5c). In consistence with previous description^45^, multiple proteins that have been commonly found in exosomes such as hnRNPA2B1, PARK7, RPS3, ENO1, EEF2, and PKM were detected in our exosomal proteomic data (Supplementary Data 3). According to the genes that were detected in both transcriptome and proteome data, the expression correlations between RNAs and proteins were relatively lower in exosomes than in their parental cells (Supplementary Fig. 7b). In comparison with the correlation analysis results of transcriptome data, the proteomic data showed about a threefold increase in correlations between exosomes and their parental cells (Fig. 5a, c). The results suggested that the expression profiles were different between exosomal RNAs and proteins, and exosomes exhibited a closer proxy of their parental cells at the proteomic level.

### High concordance of the highly expressed genes between exosomes and their parental cells

Although the weak correlations of overall gene expression levels between exosomes and their parental cells were observed as shown above, we hypothesized that there may be a subset of genes exhibiting the high concordance. To test this hypothesis, we applied hypergeometric tests to evaluate the significance of representation of highly and lowly expressed cellular contents in exosomes. Among the top 5% of expressed protein-coding genes (totally 997 genes) in LSLCs, 46% (460/997) were listed in the top 5% of most highly expressed genes in LSLC-EXO (Fig. 5d). Consistently, there were 30% (296/997) of weakly expressed protein-coding genes (the bottom 5% of expressed genes) shared between LSLC and LSLC-EXO (Fig. 5d). We further analyzed the top 10, 15, and 20% of most highly or lowly expressed genes, and similar patterns were observed (Supplementary Fig. 7c). Consistent results were also obtained for lncRNA genes and between LBC and LBC-EXO (Supplementary Fig. 7d). In contrast, only 4.5% genes were shared between cellular and exosomal samples for random analysis. Next, we further examined the observations using proteomic data. Consistent with transcriptomic analysis, high concordances of the most highly expressed proteins between exosomes and their parental cells were observed (Fig. 5d); however, the weakly expressed proteins exhibited similar patterns to random selections. These results suggested that the transcripts or proteins that are highly expressed in LSLCs or in LBCs were more prone to be highly expressed in their derived exosomes.

To further investigate the expression patterns of the highly expressed genes of exosomes in other sample types, we performed comparison analysis using transcriptomic and proteomic data from ExoRbase^46^ and ExoCarta^47^ database. Briefly, an average of 34% (336/997) and 32% (320/997) of highly expressed genes (top 5%) were shared between LSLC(LBC)-EXO and exosomes of hepatocellular carcinoma (HCC) or colon cancer, respectively. Several biological functions such as the signal recognition particle pathway, cell–cell adhesion, and cell movement were significantly enriched in these common highly expressed genes (Supplementary Data 4). Among the top 100 frequently expressed proteins in exosomes collected by ExoCarta, 39 proteins were listed in the top 5% of most highly expressed proteins in our study (Supplementary Data 4). These results suggested that a subset of genes was highly expressed in exosomes across various cell types and may play important roles in the regulation of exosome biogenesis.

### Functional characterization of exosomal-enriched genes at the transcriptomic and proteomic levels

**Fig. 6:**
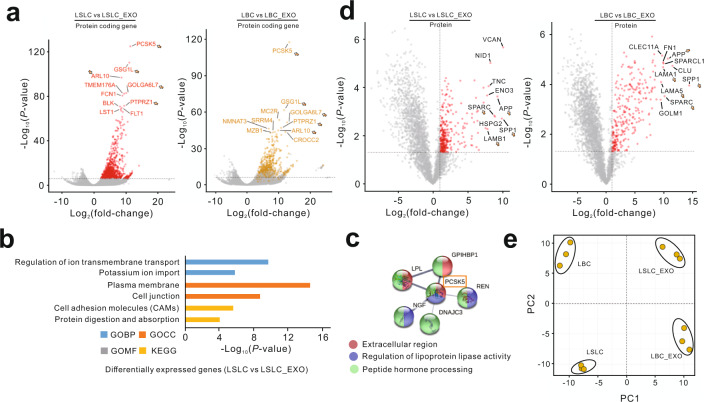
Identification and functional characterization of exosomal-enriched RNAs and proteins. **a** Volcano plots showing exosomal-enriched protein-coding genes identified using transcriptomic data. Red dots denote the genes passing our *P* value and fold difference thresholds. Yellow dots in the right panel denote the LSLC-EXO-enriched genes (the red dots in the left panel). The genes listed in the top 10 significance are marked with gene names. Arrows denote the common genes between cells and exosomes. **b** Functional annotation of differentially expressed genes between LSLC and LSLC-EXO. **c** The PPI subnetwork of PCSK5 obtained from the STRING database. The significantly enriched gene annotations are listed in the bottom and colored. **d** Volcano plots showing exosomal-enriched proteins identified using proteomic data. **e** Principal component analysis based on LSLC-EXO-enriched proteins.

Next, we conducted systematic analysis to determine which genes were specifically enriched in exosomes and what functions they might serve. In comparison with LSLCs, a total of 852 protein-coding genes and 435 lncRNA genes were found to be significantly enriched in LSLC-EXO (Fig. 6a, Supplementary Fig. 8a, and Supplementary Data 5). In agreement with this data, more than half of the listed protein-coding genes (482/852) and lncRNA genes (252/435) were also found to be significantly highly expressed in LBC-EXO relative to LBC (Supplementary Fig. 8b–d and Supplementary Data 5). Based on Gene Ontology (GO) biological processes analysis, these genes were significantly enriched in ion transport and import (*P* value = 1.84e−10), which was consistent with the transporting roles of common exosomes (Fig. 6b, Supplementary Fig. 9a, and Supplementary Data 6). When looking at GO cellular components, we found that these exosomal-enriched genes were mainly localized to plasma membrane and cell junction categories. Furthermore, according to Kyoto Encyclopedia of Genes and Genomes (KEGG) pathway enrichments, “cell adhesion molecules” (*P* value = 2.26e−6) and “protein digestion and absorption” (*P* value = 8.43e−5) pathways were significantly enriched in exosomes. Among the top 10 most enriched genes, 5 genes were common between LSLC-EXO and LBC-EXO, including *PCSK5*, *GSG1L*, *ARL10*, *GOLGA6L7*, and *PTPRZ1* (Fig. 6a). Specifically, as the most significant gene, *PCSK5* as well as its interacted genes were localized to the extracellular region and associated with “regulation of lipoprotein lipase activity” and “peptide hormone processing” (Fig. 6c).

In comparison with intracellular proteomic profiles, we identified 302 and 272 proteins that were highly expressed in LSLC-EXO and LBC-EXO, respectively, 74 of which were common (Fig. 6d and Supplementary Data 7). It has previously been reported that cancer cell-derived exosomes as efficient carriers in mediating molecular exchange contained a rich cargo of proteins with diverse functions in tumor progression^28^. Consistently, in addition to proteins involved in exosome biogenesis, we found that exosomes contained numerous proteins related to tumorigenesis and metastasis, such as cell adhesion, integrin binding, and ECM–receptor interaction (Supplementary Fig. 9b, c and Supplementary Data 8). As the highly expressed protein with the largest fold change in both LSLC-EXO and LBC-EXO, SPP1 could interact with several proteins, most of which were involved in ECM organization (Supplementary Fig. 9d). Notably, several exosomal integrins (including ITGAV, ITGA9, ITGB1, and ITGB5) that have been found to be upregulated in lung cancer and could be used to predict tumor metastasis^48^ were connected with the SPP1 protein. Moreover, according to the protein–protein interaction (PPI) networks of the most exosomal-enriched proteins, such as SPARC, FN1, HSPG2, APP, and several members of the laminin family, we found that exosomes might be involved in regulation of cell migration and the apoptotic process (Supplementary Fig. 9e). In addition, based on proteomic profiles of LSLC-EXO-enriched proteins, the distinct clusters among LSLCs, LBCs, and their derived exosomes were observed using principal component analysis (PCA) (Fig. 6e), suggesting the unique expression patterns of subset of proteins in LSLC-EXO. Notably, there was almost no overlap between exosomal-enriched RNAs and proteins, suggesting the distinct patterns of exosomes at the transcriptomic and proteomic levels (Supplementary Data 5 and 7).

### Potential LSLC markers could be transferred by LSLC-EXO

**Fig. 7:**
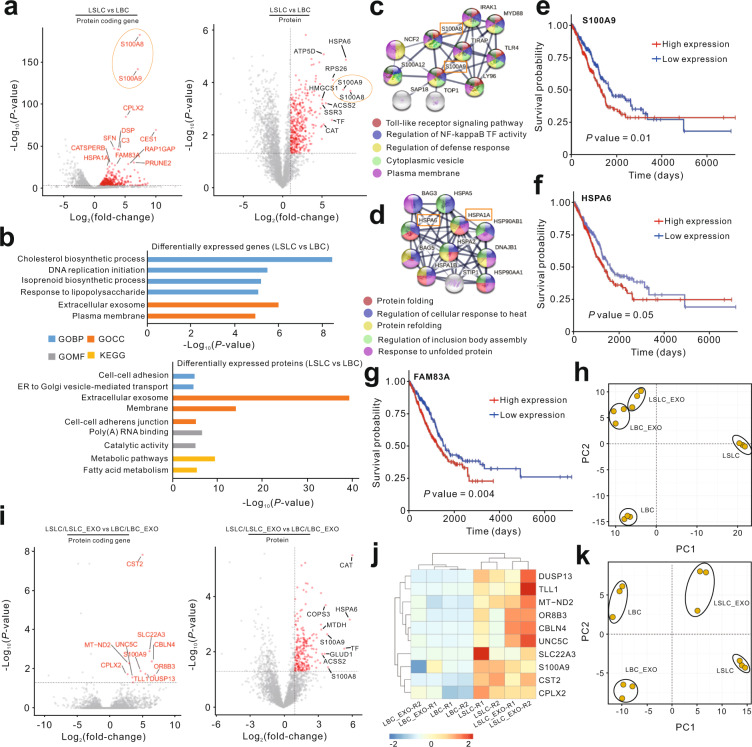
Functional and clinical significance of LSLC markers. **a** Volcano plots showing LSLC markers identified using transcriptomic (left panel) and proteomic (right panel) data. Red dots denote the genes passing *P* value and fold difference thresholds. The common genes between transcriptomic and proteomic data are circled. **b** Functional annotation of LSLC markers from transcriptomic (top panel) and proteomic (bottom panel) data. **c**, **d** The PPI subnetwork of representative LSLC markers obtained from the STRING database. The significant enriched gene annotations are colored. **e**–**g** The survival curves based on TCGA LUAD data showing that patients with higher expression of LSLC markers had poor prognosis. **h** Principal component analysis based on LSLC marker proteins. **i** Volcano plots showing significantly highly expressed genes in LSLC and LSLC-EXO based on transcriptomic (left panel) and proteomic (right panel) data. **j** Heatmap showing the significantly highly expressed genes in LSLC and LSLC-EXO at the transcriptomic level. **k** Principal component analysis based on significantly highly expressed proteins in LSLC and LSLC-EXO.

Exosomes released by CSCs may carry cancer recurrence or therapy resistance biomarkers, which could be used as potential targets for cancer diagnosis and prognosis. Based on multi-omics data generated in this study, we performed further analysis to identify and characterize the LSLC-specific markers and determine which of them could be transferred by exosomes. First, the results showed that the expression levels of 385 genes at the RNA level and 353 proteins were significantly higher in LSLCs than in LBCs and thus referred to as potential LSLC markers (Fig. 7a and Supplementary Data 9). Several cancer-related pathways were enriched for these, including the cholesterol biosynthetic process, cell–cell adhesion, and metabolic pathways (Fig. 7b and Supplementary Data 10). Although we have demonstrated the weak correlations and different expression profiles between transcriptomic and proteomic data, relative concordance between them was observed that was related to the identification of LSLC markers. For example, calcium-binding proteins S100A8 and S100A9 that have been found to be upregulated in multiple cancer types^49^ were highly expressed in LSLCs compared with LBCs. The PPI network of S100A8 and S100A9 is associated with several regulatory functions, such as the toll-like receptor signaling pathway, regulation of nuclear factor-κB transcription factor activity, and regulation of defense response (Fig. 7c). In addition, the members of the heat shock protein family, including HSPA1A and HSPA6, whose functions were associated with protein folding/refolding and cellular response^50^, were also identified as LSLC markers at the multi-omics level (Fig. 7a, d). According to The Cancer Genome Atlas lung adenocarcinoma (TCGA-LUAD) data, we found that multiple LSLC markers such as S100A9, HSPA6, and FAM83A were associated with a poor prognosis in lung adenocarcinoma patients (Fig. 7e–g). PCA analysis based on LSLC markers exhibited the distinct clusters between LSLCs and LBCs (Fig. 7h).

The intercellular communications between LSLCs and LBCs could be partially mediated by exosomes. We reasoned that the LSLC markers that were relatively abundant in LSLC-EXO, while not or lowly expressed in LBC and LBC-EXO, could server as mediators between LSLCs and LBCs. These LSLC-EXO-transported LSLC markers could also act as liquid biopsy biomarkers for early detection and diagnosis of lung adenocarcinoma. To identify such LSLC markers, we evaluated the expression patterns of all LSLC markers at both the transcriptomic and proteomic levels across all samples. In comparison with LBC and LBC-EXO, 218 LSLC markers were highly expressed in LSLCs and LSLC-EXO (Fig. 7i and Supplementary Data 11). For example, S100A9, which has been identified as a CSC marker and associated with poor survival^51^, was specifically expressed in LSLCs and LSLC-EXO (Fig. 7i, j). Moreover, distinct clusters between LSLCs and LBCs as well as between LSLC-EXO and LBC-EXO were observed according to PCA analysis of LSLC markers that could be packaged into LSLC-EXO (Fig. 7k). Taken together, we identified a list of potential LSLC markers with diverse functions that could be transferred by LSLC-EXO.

### Validation of LSLC markers in tumor tissues of lung adenocarcinoma patients

**Fig. 8:**
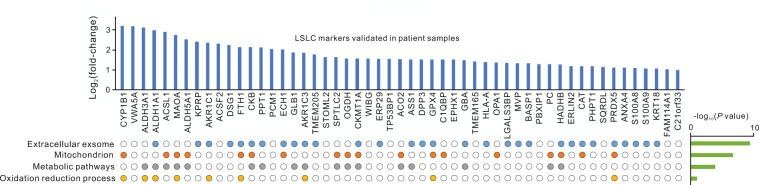
The LSLC markers that are validated in patient samples. The gene annotations and their enrichment *P* values are shown at the bottom.

To determine the clinical significance of identified LSLC markers, we performed experimental validation using patient samples. First, three tumor tissues with histopathological diagnosis as invasive lung adenocarcinoma type were collected and were further digested into single cells. Then the cells with high ALDH activity were isolated from the first passage of primary lung adenocarcinoma cells and named as patient-derived LSLCs (PLSLCs; Supplementary Fig. 10a). The results showed that the levels of stem-like genes, NANOG and OCT4, were significantly higher in ALDEFLUOR-positive cell populations than in ALDEFLUOR-negative or bulk cell populations (Supplementary Fig. 10b). Next, proteomic profiling of PLSLC and primary lung adenocarcinoma cells from patient 1 and patient 2 were analyzed using the SISPROT method and desalted for MS analysis. The cells from patient 3 did not meet the quality control for proteomic profiling and thus was excluded in the following analysis. An average of 1852 proteins were identified for each patient (Supplementary Data 12). According to the results of comparative analysis, a total of 53 LSLC markers were validated based on proteomic data of patients (Fig. 8 and Supplementary Data 13), which were more highly expressed in PLSLC than when compared with primary lung adenocarcinoma cells. Consistent with experimental analysis, ALDH proteins, such as ALDH3A1, ALDH1A1, and ALDH5A1, were listed as the top 10 most changed proteins. These validated LSLC markers were significantly enriched in extracellular exosome and involved in several bioterms, including metabolic pathways and oxidation reduction processes (Fig. 8). Their clinically relevant roles in the diagnosis of lung adenocarcinoma need further investigation.

## Discussion

As a research hotspot over the past decade, studies on cancer-derived exosomes have expanded our understanding of the cross-talk among tumor cell subpopulations or between tumor cells and their microenvironments^13,19,35^. In spite of this progress, many fundamental questions about the biological functions of exosomes involved in tumor ecosystems remain and need to be addressed, mainly due to the extensive intratumor heterogeneity. According to the CSC hypothesis^6^, a tumor contains a CSC subpopulation that exhibits key stem cell capacities, including self-renewal capacity, that drives tumorigenesis and differentiation capacity that contributes to tumor heterogeneity. However, there is very little known about the exosomes released by such a CSC subpopulation. In this study, we provide the multi-omics analysis of exosomes released by LSLCs and corresponding bulk cancer cells.

Since the first experimental evidence for theories of CSC was provided in human leukemias in 1997^52^, CSCs have been identified and extensively studied in a variety of cancer types, such as breast cancer^21^, melanoma^22^, pancreatic cancer^53^, and prostate cancer^54^, yet, the definitive CSC markers for specific cancer types are still highly debated. In the current study, we utilized 3D culture system, which has been widely used to study cell proliferation and migration^55^, to conduct the cell line experiments. According to our tumor sphere formation results, the self-renewal capacity of CSCs was effectively evaluated based on 3D culture system. By using in vitro and in vivo assays, we demonstrated that ALDEFLUOR-positive cells exhibit phenotypic and functional traits of CSCs, including self-renewal, differentiation, and high proliferation capacities, and are able to generate tumors in a xenotransplantation animal model. This indicates that ALDH activity could be used as a marker for CSC isolation from lung adenocarcinoma cells.

The exosomes released by LSLCs and LBCs were successfully isolated using multi-step ultracentrifugation that has been regarded as the most commonly employed exosomal isolation approach. The shape and the size distribution of isolated exosomes were confirmed by TEM and NAT. The specific exosomal markers CD9, CD63, and CD81 further confirmed the presence of exosomes. According to the data of Ribo-zero RNA-seq, the RNA compositions of exosomes and their parental cells are highly distinct. Both LSLC-EXO and LBC-EXO exhibited selective RNA packaging compared with their parental cells, with lncRNAs and intronic regions being more enriched in exosomes. Notably, for lncRNA genes, the percentage of reads mapping to exonic and intronic regions were different between LSLC-EXO and LBC-EXO (Supplementary Fig. 5c), with the larger proportion of exonic reads in LSLC-EXO, which may reflect the differential release pathways for exonic and intronic sequences of lncRNAs between LSLCs and LBCs. Consistent with previous findings^28^, fragmented transcripts constituted the majority of RNA repertoires in exosomes. The fragmental events involved in exosomes may also contribute to the minimal presence of circRNAs compared with cells. We speculate that the exosomal RNA fragments may carry important functional elements and thus were selectively packaged into exosomes. Moreover, the analysis results of RNA variants showed that the portions of synonymous variants between LSLC_EXO and LBC_EXO (35 and 56%, respectively) were different (Fig. 4c), which may either reflect the difference in their intrinsic RNA processing events or due to the technical noise. Generally, RNA variants may be generated from DNA alterations or RNA editing events; it was hard to distinguish them in current study. Further studies to uncover these underlying mechanisms are needed.

To gain a systematic and comprehensive understanding of the tumor stem cell-derived exosomes, we conducted the combined analysis using both transcriptomic and proteomic data. Weak correlations were observed either between these two data types or between exosomes and their parental cells. In such a situation, we found that exosomes more closely reflect their parental cells at the proteomic levels. Considering that the expression patterns of genes or proteins in cellular space may influence their encapsulation into exosomes, we compared the levels of RNA and protein contents between exosomes and their parental cells. The results indicated that highly expressed genes or proteins in cells were more abundant in their released exosomes. The biological functions of exosomes in lung adenocarcinoma are largely unknown. Our data suggested that multiple ion transport genes and cell–cell communication genes were packaged into exosomes. Furthermore, we found that several functional programs associated with the clonal events such as cell proliferation, cell adhesion, and cell migration were involved in the CSCs and their exosomes. These genes provide a new resource for investigating the mechanisms of exosomal regulations in cancer.

Previous studies demonstrated that CSC-derived exosomes could influence resident tumor cells or remodel the tumor microenvironment^19,28^, which could induce a stemness phenotype and lead to tumor development and metastasis. In lung adenocarcinoma, differential RNA and protein patterns were observed in LSLCs and LBCs. Numerous highly enriched genes in LSLCs known to contribute to toll-like receptor signaling pathway, metabolic pathways, and cell–cell adhesion could be transferred by exosomes. These findings will guide future development of exosomal biomarkers for cancer diagnosis.

Overall, the key outcome of our study provides the detailed description of the constituents and functional properties of exosomes released by LSLCs at the multi-omics level. The findings in this current study open new avenues toward understanding the biogenesis and biological functions of tumor-derived exosomes and thus facilitate the clinical application of CSCs and their exosomes. In particular, the potential LSLC markers transferred by exosomes require further experimental studies to explore their regulatory mechanisms as well as verify their clinical significance.

## Methods

### Lung adenocarcinoma cell lines

The human non-small cell lung cancer (NSCLC) cell lines A549, H1734, and H1975 were obtained from the American Type Culture Collection. The cells were cultured in Dulbecco’s modified Eagle’s medium (DMEM) supplemented with 10% fetal bovine serum (FBS), Glumax-1, and sodium solution at 37 °C under a humidified atmosphere consisting of 5% CO_2_. In addition, cells were cultured in DMEM with 10% free-exosome FBS for 72 h in order to extract the exosomes. The free-exosome FBS was harvested by ultracentrifugation at 120,000 × *g* (Type 45 Ti rotor) for 18 h.

### Primary culture of lung adenocarcinoma cells from patients

Fresh lung adenocarcinoma tissues were obtained from three patients who were pathologically diagnosed with NSCLC. Written informed consent was provided by all patients. Tissue samples were immediately saved in sterile medium with 1% penicillin–streptomycin after surgical resection and transported to the laboratory on ice within 30–60 min of removal. Then the samples were rinsed with phosphate-buffered saline (PBS) three times and the peritumoral tissues containing necrotic tissue and nonmalignant lung tissue were removed with sterile scissors under sterile conditions. Next, the remaining tumor tissues were mechanically sliced into about 1 mm^3^ pieces by ophthalmic scissors and tweezers. Then the pieces underwent enzymatic digestion as per the protocol of Primary Tumor Cell Isolation Kit (IMMORTECH, China). The tumor fragments were incubated into enzymatic solution provided by the kit and placed for 2 h at 37 °C. Digestion of the samples was terminated and then passed through 70 μM nylon cell strainers (Falcon, BD), then centrifuged for 5 min at 300 × *g* at room temperature and the precipitates were resuspended by 1× red blood cell (RBC) lysis buffer (Santa Cruz Biotechnology, TX, USA) to lysis erythrocytes for 10 min because of the large amount of RBCs in the tumor tissues. The remaining cells were washed twice in PBS and then resuspended in primary tumor cells culture media (IMMORTECH, Guangdong, China). LIVE/DEAD cells were counted by use of AO/PI staining solution on cellular counter (Cellometer, USA). All experiments using patient samples were carried out according to the regulation set by the Institutional Ethics Committees at Shenzhen People’s Hospital (Shenzhen, China).

### ALDEFLOUR assay

The ALDEFLOUR Assay Kit (Stemcell Technologies, BC, Canada) was used to measure ALDH activity of cell lines and primary tumor cells as per the manufacturer’s protocol. Cells were treated with ALDEFLOUR assay buffer containing activated ALDEFLOURTM Reagent BODIPY-aminoacetaldehyde and analyzed by fluorescence-activated cell sorter (FACS) instrument (Sony). As negative control, cells were treated with diethylaminobenzaldehyde, which is a special ALDH inhibitor and analyzed by FACS sorter instrument (Sony).

### Tumor sphere culture

The tumor spheres were cultured in the serum-free suspension 3D culture system mixing spheroid culture medium and matrigel (1:1). The spheroid culture medium contained DMEM/F12K (1:1) medium supplemented with 20 ng/mL epidermal growth factor, 20 ng/mL basic fibroblast growth factor, 5 µg/mL insulin, 1% penicillin–streptomycin, and 1% B27 supplement. The tumor spheres would be formed with 5–7 days and should be passaged once every 7 days. When passaged for the next generation, the spheroids were digested with accutase enzyme for 15–30 min at 37 °C and centrifuged at 1000 rpm for 5 min. The images of forming spheres were captured using the microscope (Olympus, Japan) at ×100 magnification.

### Real-time quantitative PCR (qPCR) detection

Total RNA was extracted with Trizol and 1 μg of total RNA were reverse-transcribed into cDNA using the Primescript^TM^ RT Reagent Kit (Takara, Shiga, Japan). By using a StepOne Plus real-time PCR system (Applied Biosystems) and SYBR Green Kit (TAKARA, Shiga, Japan), real-time reverse transcriptase q-PCR was conducted to observe the expression levels of stem genes—Nanog (5’-AAGAGGTGGCAGAAAAACAACT-3’) and OCT4 (5’-GGGGTTCTATTTGGGAAGGTAT-3’). In addition, GAPDH (5’-AGAAGGCTGGGGC TCATTTG-3’) was used as the internal control. The primers were synthesized by Sangon Biotech. All target-gene expression levels were normalized to GAPDH and defined as −∆CT[−∆CT = −(CT_targe_ − CT_GAPDH_)]. When calculating the relative expression ratio, the fold change was relative to the control (2^−∆∆CT^).

### Flow cytometric analysis

The stem-like characterization of cells was detected using flow cytometer (FC500-MPL, Beckman). The following monoclonal antibodies were used for staining: allophycocyanin (APC) mouse IgG1, κIsotype Control (5 µL/test, 5400120, Biolegend, CA, USA), fluorescein isothiocyanate (FITC) mouse IgG1, κIsotype control (5 µL/test, 400108, Biolegend, CA, USA), anti-human CD24-FITC (5 µL/test, 311104, Biolegend, CA, USA), and anti-human CD44-APC (5 µL/test, 338806, Biolegend, CA, USA). In all, 1 × 10^6^ single-cell suspension was resuspended in 300 µL PBS with 3% FBS and stained with monoclonal antibodies for 15 min in the dark at room temperature. Then cells were resuspended with PBS containing 3% FBS and analyzed using a flow cytometer.

### Colony formation assay

In order to observe the cell’s ability of forming colonies, the colony formation assay was tested. Single-cell suspensions were planted at a concentration of 2000 cells per well of 6-well tissue culture plates pre-treated with matrigel in DMEM, supplemented with 1% penicillin–streptomycin and 0.1% FBS, and cultured for 7–10 days in a 5% CO_2_ incubator at 37 °C. The culture media were replaced every other day. After 10 days, the plates were taken out from the incubator and the growth media were aspirated. The cell monolayer was washed with PBS twice and then fixed with 4% paraformaldehyde for 20 min. The cell monolayer was washed and stained with 500 µL Crystal Violet staining solution at room temperature for 10 min. The solutions were aspirated carefully, and cell monolayer was washed several times until the plates became clear. The culture plates were dried at room temperature and the colonies consisting of >50 cells were counted.

### Xenograft tumor formation assay

Equal numbers (100, 1000, and 10,000) of sorted ALDH1^−^ cells and ALDH1^+^ cells were suspended in 150 µL PBS-matrigel (1:1) and subcutaneously injected into parallel sites (ALDH^−^ in the left flank while ALDH^+^ in the right flank) of 4–7-week-old female Balb/c nude mice. After injection, the tumor formation was recorded, and the weight of mice was measured every 2 days. All experiments using animal models were carried out according to the regulation set by the Institutional Ethics Committees at Shenzhen People’s Hospital (Shenzhen, China).

### Isolation and identification of exosomes using ultracentrifugation

The culture media was harvested and then centrifuged sequentially (400 × *g* for 5 min; 2000 × *g* for 20 min) to remove floating and dead cells. The supernatant was further centrifuged at 10,000 × *g* for 30 min to remove cell debris and filtered through 0.22 µm Millipore filter. Then the exosomes were extracted from the cleared and filtered supernatant by ultracentrifugation at 150,000 × *g* for 2 h at 4 °C. The supernatant was discarded and the bottom pellet was washed with ice-cold PBS containing 0.1% tween-20, ultra-centrifugated again at 150,000 × *g* for 2 h at 4 °C. Then the supernatant was discarded and the bottom pellet was washed with ice-cold PBS, ultra-centrifugated again at 150,000 × *g* for 2 h at 4 °C. The pellets were resuspended with ice-cold PBS and conserved at −80 °C for further study. The concentration of exosomal proteins was quantified by the BCA Protein Assay Kit (Thermo Scientific).

### Western blot analysis

Cells and exosomes were lysed in RIPA lysis buffer containing protease inhibitors (89901, Thermo Fisher Scientific, MA, USA) for 20 min on ice. The lysed proteins were quantified and loaded for electrophoresis and electrotransfer. The antibodies CD63 (1:1000, ab134045, Abcam, Cambridge, UK), CD9 (1:2000, ab92726, Abcam, Cambridge, UK), CD81 (1:1000, ab109201, Abcam, Cambridge, UK), OCT4 (1:1000, ab19857, Abcam, Cambridge, UK), and NANOG (1:1000, ab109250, Abcam, Cambridge, UK) were incubated according to the instruction. All blots or gels were derived from the same experiment and were processed in parallel. Un-cropped images of all blots are shown in Supplementary Figs. 11 and 12.

### TEM and NTA

Exosome shape was verified using transmission electron microscope JEM-1230 (Nippon Tekno). The suspensions containing exosomes were blown by pipette tip and loaded 20 µL to copper grids. When the copper grids were dry, they were stained with 1% uranyl acetate solution, then observed using transmission electron microscope (HT7700, NIPPON TEKNO, Japan). Suspensions containing exosomes were analyzed using the Nanoparticle system (Nanosight NS300, UK) to observe the exosome size and number.

### RNA isolation and Ribo-zero RNA-seq

In this study, 3–5 × 10^6^ cells were used for the isolation of exosomes. In all, 10–15 and 30–50 µg LSLC-EXO and LBC-EXO, respectively, were obtained. At least 10^6^ cells were used for Ribo-zero RNA-seq. Total RNA of cellular and exosomal samples were isolated using TRIzol™ reagent (Invitrogen, Carlsbad, CA, USA) according to the manufacturer’s instructions. The concentrations of RNA were quantified using the Qubit® RNA Assay Kit in Qubit® 2.0 Flurometer (Life Technologies, CA, USA). RNA purity and integrity were checked using the NanoPhotometer® spectrophotometer (IMPLEN, CA, USA) and the RNA Nano 6000 Assay Kit of the Bioanalyzer 2100 system (Agilent Technologies, CA, USA), respectively. rRNA was removed by Epicentre Ribo-zero™ rRNA Removal Kit (Epicentre, USA). rRNA-free residue was cleaned up by ethanol precipitation. Then RNA-seq libraries were constructed using the NEBNext® Ultra™ Directional RNA Library Prep Kit for Illumina (NEB, USA). The libraries were sequenced on the Illumina Hiseq 4000 platform with 150 bp paired-end reads.

### RNA-seq raw data quality control and reads mapping statistics

Raw sequencing data (raw reads) from Illumina Hiseq 4000 sequencer was processed to filter out low-quality reads and the reads containing ploy-N and adapter sequence. Clean reads from each sample were aligned to the human reference genome (version hg38/GRCh38) by using STAR aligner^33^ (v2.7.1) (set the *twopassMode* as Basic). The BAM files (mapping results) were sorted and indexed using samtools^56^.

### Gene expression ratio statistics

The reference gene models were downloaded from the GENCODE database (version 35) (https://www.gencodegenes.org/human/). The read counts and transcripts per million of reference genes were calculated using pseudoalignment tool Kallisto^30^ (v0.46.0). Gene expression level was summarized from the transcript level. For each gene type annotated by the GENCODE database (v35), the proportion of expressed genes was obtained based on the number of expressed genes (the read counts more than one) divided by the total number of genes of the gene type. The information of 3’UTR, 5’UTR, CDS, and intron regions of protein-coding genes as well as the exon and intron regions of lncRNA genes were obtained using Table Browser tool of the UCSC Genome Browser (http://genome.ucsc.edu/cgi-bin/hgTables). Based on indexed BAM files and region files, the mapping statistical result for each region was calculated using bedtools^57^. The distribution and coverage analysis of mapped reads across gene body was conducted by RSeQC^58^ package.

### Transcripts assembly and evaluation

Based on read alignment results outputted by STAR aligner, StringTie^32^ (v2.0.3) was used to de novo assemble transcripts (reference gene models were not used to guide the assembly process). The assemble transcripts were compared with reference gene models using the Cuffcompare utility provided by the Cufflinks^59^ package. The reference-matched transcripts were then classified into three categories according to the “class code” reported by Cuffcompare, including complete match (class code is “=”), partial match (class code is “j”), and contain (class code is “c”).

### Variants’ calling

After read alignments, PCR duplicates were marked using the MarkDuplicates module of GATK^60^. Then RNA sequence variants were called using the HaplotypeCaller algorithm of GATK. The obtained VCF files of samples were annotated using the vcf2maf tool (https://github.com/mskcc/vcf2maf).

### circRNA identification

Based on read alignment results and reference gene models, CIRIquant^42^ was applied for circRNA identification. Briefly, the spliced reads were filtered out and the unmapped reads were used for circRNA detection. The back-spliced junction sites were identified by CIRI2^61^. Then the pseudo-circular reference sequences were generated by concatenating two full-length sequence of the back-spliced junction region. The candidate circular reads were aligned to the pseudo-circular sequence again. The circular splice junction reads were determined according to the re-alignment result. The identified circRNAs that matched to the reference circRNAs^43^ (circAltas 2.0 database) were retained.

### Overlap analysis of highly expressed genes

The highly expressed gene sets were obtained according to the top 5% of most highly expressed genes. The intersection of different gene sets was visualized by Venn plot (http://cran.r-project.org/package=vennplot). The significance of overlaps was assessed using the hypergeometric test. Transcriptomic data of exosomes of HCC and colorectal cancer (CRC) was downloaded from ExoRbase^46^ including 12 HCC samples and 21 CRC samples. For each dataset, the mean expression value of genes was used to get highly expressed genes. The top 100 frequently expressed proteins in exosomes were downloaded from ExoCarta^47^ database.

### Gene differential expression, function annotation, and survival analysis

Gene differential expression analysis were performed using DESeq2^62^ with default parameters. The genes with log2FoldChange >1 and with adjusted *P* value < 0.05 and with the significance ranked in the top 0.05 percentile were regarded as significantly enriched genes. Heatmaps of significantly enriched genes were generated using the pheatmap package (https://cran.r-project.org/web/packages/pheatmap/index.html). GO and KEGG analysis of the significantly enriched genes was performed using DAVID^63^ (https://david.ncifcrf.gov) with default parameters. The terms with enrichment *P* value <0.01 were retained. The PPI networks of specific proteins were established by STRING database (https://string-db.org). The TCGA-LUAD data were used to test the correlation of gene expression and patient survival (Kaplan–Meier analysis based on log-rank test), which was performed on UCSC Xena Browser (https://xenabrowser.net).

### Proteomic sample preparation

The cells or exosomes were lysed immediately with the compatible lysis buffer containing 10 mM HEPES, pH 7.4, 150 mM NaCl, 2 mM CaCl_2_, 2 mM MgCl_2_, 600 mM guanidine HCl, 1% DDM, and protease inhibitor mixture (1 mM EDTA, 1 mM phenylmethanesulfonylfluoride, 1 μg/mL leupeptin, 1 μg/mL pepstatin, and 1 μg/mL aprotinin). Protein concentration was determined by the Pierce Micro BCA Kit (Thermo). The obtained tissue lysate was processed by using the SISPROT protocol as previously described. Briefly, the samples were first acidified to pH 2–3 and loaded onto 200 or 10 μL spin-tip device packed with one plug of C18 disk (3M Empore, USA) and 0.6 mg of 20 μm POROS SCX beads (Applied Biosystems, USA) in tandem. Proteins were reduced by TCEP, alkylated by IAA and digested by trypsin (TPCK-treated, Sigma-Aldrich). The digested peptides were then transferred from the SCX beads to C18 disk with 200 mM ammonium formate (pH 10) and eluted from C18 disk with ACN concentration of 80% in 5 mM ammonium formate (pH 10).

### LC-MS/MS analysis

The obtained samples were resuspended in 0.1% (v/v) formic acid (FA) and analyzed by a Q-Exactive HF-X mass spectrometer coupled with an Easy-nLC 1000 (ThermoFisher Scientific). The LC separation was performed with an integrated spray-tip column (100 μm i.d. × 20 cm) packed with 1.9 μm/120 Å ReproSil-Pur C18 resins (Dr. Maisch GmbH, Germany). The gradient solvent system consisted of solvent A [0.1% (v/v) FA in water] and solvent B [0.1% (v/v) FA in ACN]. In all, 80% (v/v) of the peptide samples were loaded and separated at a flow rate of 250 nL/min. The solvent B was changed linearly as follows: 0 min, 3%; 2 min, 7%; 52 min, 22%; 62 min, 35%; 64 min, 90%; 70 min, 90%; 72 min, 3%; 80 min, 3%. Full MS scans were performed in mass analyzer over *m*/*z* range of 350–1550 with a mass resolution of 120,000. The MS/MS spectra were acquired in data-dependent acquisition mode with a 3-s Top Speed method. Tandem MS was performed in the ion-trap mass analyzer using an isolation window of 1.6 Da by quadrupole mass analyzer and HCD fragmentation with normalized collision energy of 30. The dynamic exclusion time was set as 60 s.

### Proteomic data processing

Raw proteomic data were searched against the human Uniprot fasta database (70,332 entries, downloaded on Sep 29, 2016) using MaxQuant (version1.5.5.1)^44^ for label-free quantification (LFQ) with “match between run” function activated. The false discovery rate evaluation was done by searching a reverse database and was set to 0.01 for proteins and peptides. The following parameters were used for the LFQ analysis: cysteine carbamidomethylation was set as fixed modification, while methionine oxidation, asparagine, and glutamine deamidation were set as variable modifications for the global protein identification. The mass tolerances of precursor and fragment ions were set to 5 ppm and 0.02 Da, respectively. The maximum missed cleavages for trypsin digestion was set to 2. Other parameters were set as default. The statistical analyses were performed using the Perseus software (version 1.5.5.3)^64^. Only proteins identified with ≥2 peptides and 2 valid values in at least one group were reserved for further analysis. Missing values were assigned an artificial value sampled from a normal distribution (width = 0.3, down-shift = 1.8). The fold changes (*t* test difference, log2 ratios of the mean of the normalized LFQ ratio from three replicates) were calculated and plotted against the −log10 of the *P* values derived from *t* test. The proteins with log2FoldChange >1 and with *P* value <0.05 were regarded as significantly highly expressed proteins.

### Ethics approval

Both the human study and the animal study were approved by the Institutional Ethics Committees at Shenzhen People’s Hospital (Shenzhen, China). Written informed consent was provided by all patients.

### Reporting summary

Further information on research design is available in the Nature Research Reporting Summary linked to this article.

## Supplementary information

Supplementary Information

Supplementary Data 1

Supplementary Data 2

Supplementary Data 3

Supplementary Data 4

Supplementary Data 5

Supplementary Data 6

Supplementary Data 7

Supplementary Data 8

Supplementary Data 9

Supplementary Data 10

Supplementary Data 11

Supplementary Data 12

Supplementary Data 13

Supplementary Data 14

Reporting Summary

## Data Availability

Raw RNA-seq data has been deposited in the Sequence Read Archive (SRA) (accession number: PRJNA663998). The mass spectrometry proteomics data has been deposited to iProX database^65^ (an official member of ProteomeXchange Consortium) (iProX ID: IPX0002790000, ProteomeXchange ID: PDX023981). All processed files, including identified SNVs, circRNAs, and quantified proteins, are made available as Supplementary Data.

## Code availability

All of the software packages used in this study can be obtained from Supplementary Data 14.
