# Supplementary material for: Dissecting the multi-omics atlas of the exosomes released by human lung adenocarcinoma stem-like cells
Source: NPJ Genom Med. 2021 Jun 14;6:48. doi: 10.1038/s41525-021-00217-5 (PMC8203745; doi:10.1038/s41525-021-00217-5)
Supplement: Supplementary file 1 — Supplementary Information [file 41525_2021_217_MOESM1_ESM.pdf]

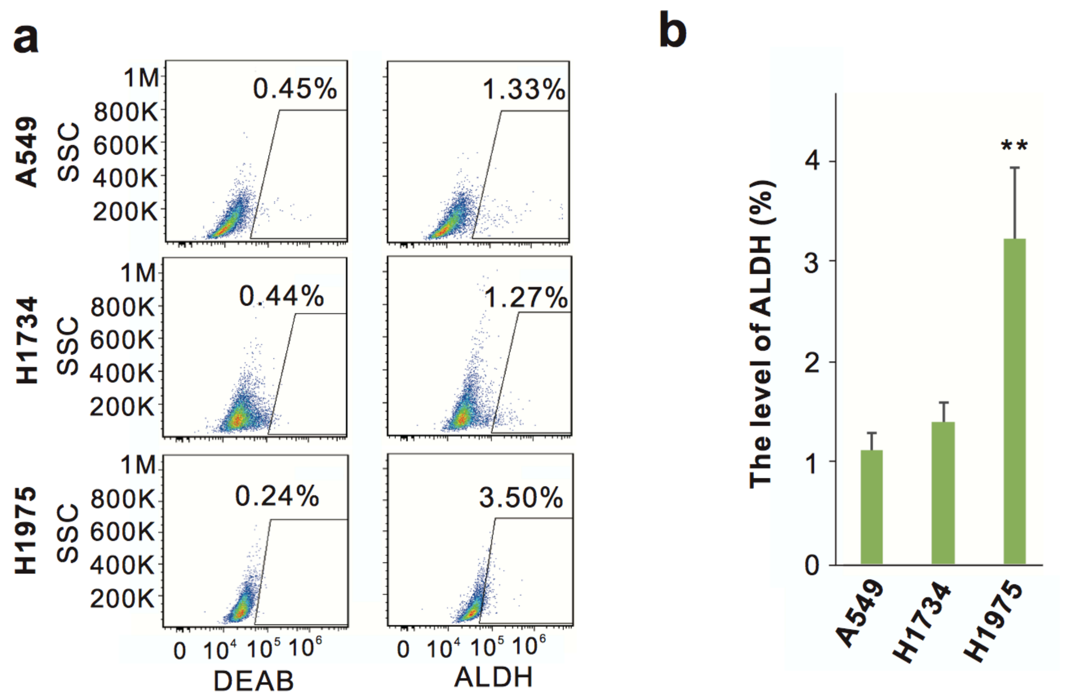

Supplementary Fig. 1. Flow cytometry analysis of ALDH activity. a Flow cytometry analysis of ALDH activity in A549, H1734 and H1975 cell lines. The specific inhibitors DEAB and ALDH were used to establish the baseline fluorescence and to define the ALDEFLUOR-positive region, respectively. **b** The proportion of ALDEFLUOR-positive cells isolated by flow cytometer. $^{**}p < 0.01$. (Error bars represent + SEM).

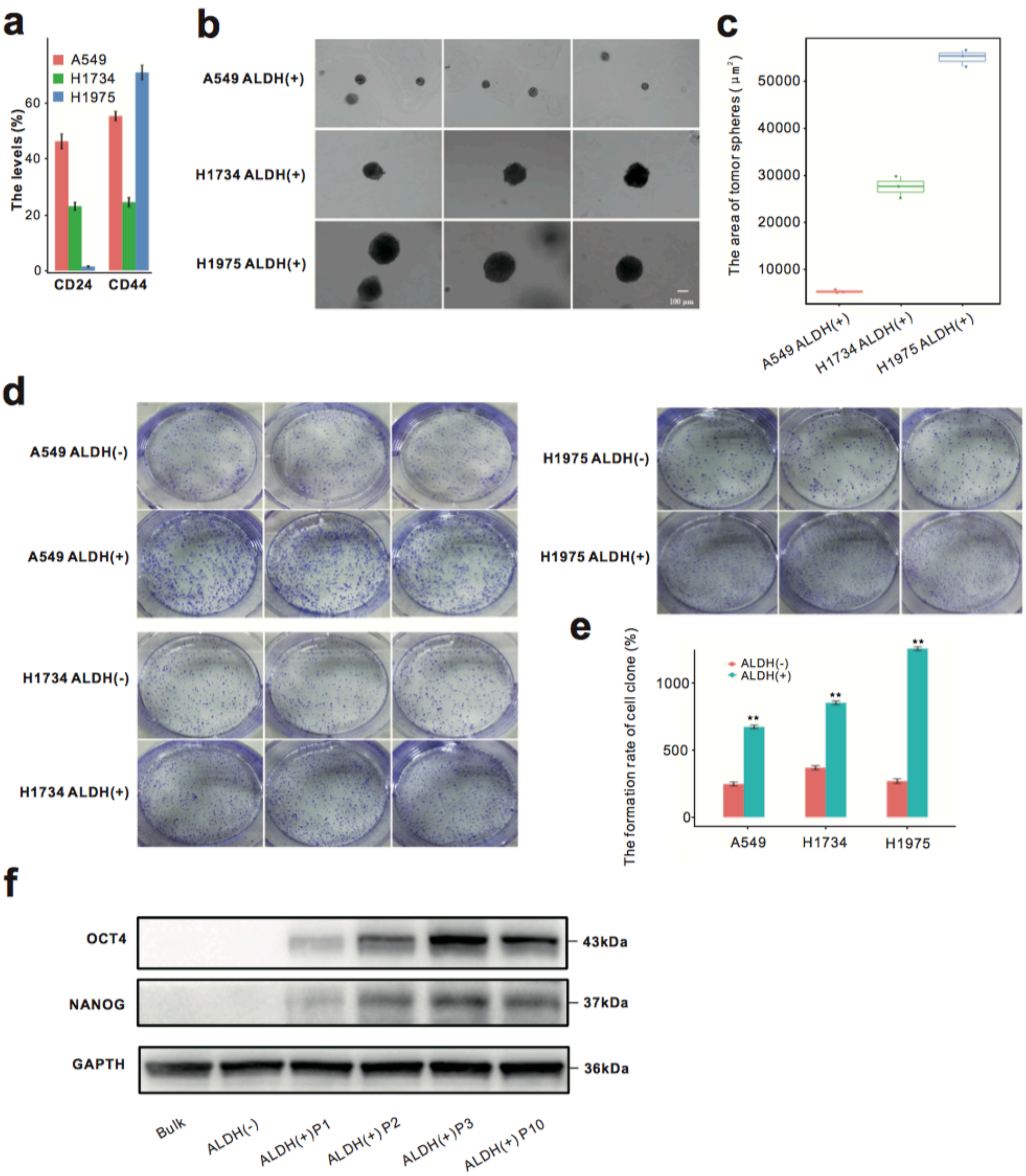

Supplementary Fig. 2. The ALDEFLUOR-positive cells from human lung adenocarcinoma have cancer stem-like cell properties. a Stem markers of lung adenocarcinoma cells were analyzed by flow cytometer using antibodies for CD24 and CD44. **b** The ALDEFLUOR-positive cells from three lung adenocarcinoma cell lines were cultured with serum-free suspension 3D culture systems for 21 days. **c** The statistics of the area of tumor spheres formed in 3D culture system for 21 days. **d, e** The view and statistics of clone formation assay of ALDEFLUOR-positive and ALDEFLUOR-negative cells. **f** The western blotting analysis of NANOG and OCT4 in bulk cells, ALDEFLUOR-negative cells, and ALDEFLUOR-positive cells with different passage from H1975 cell lines. (Error bars represent ± SEM).

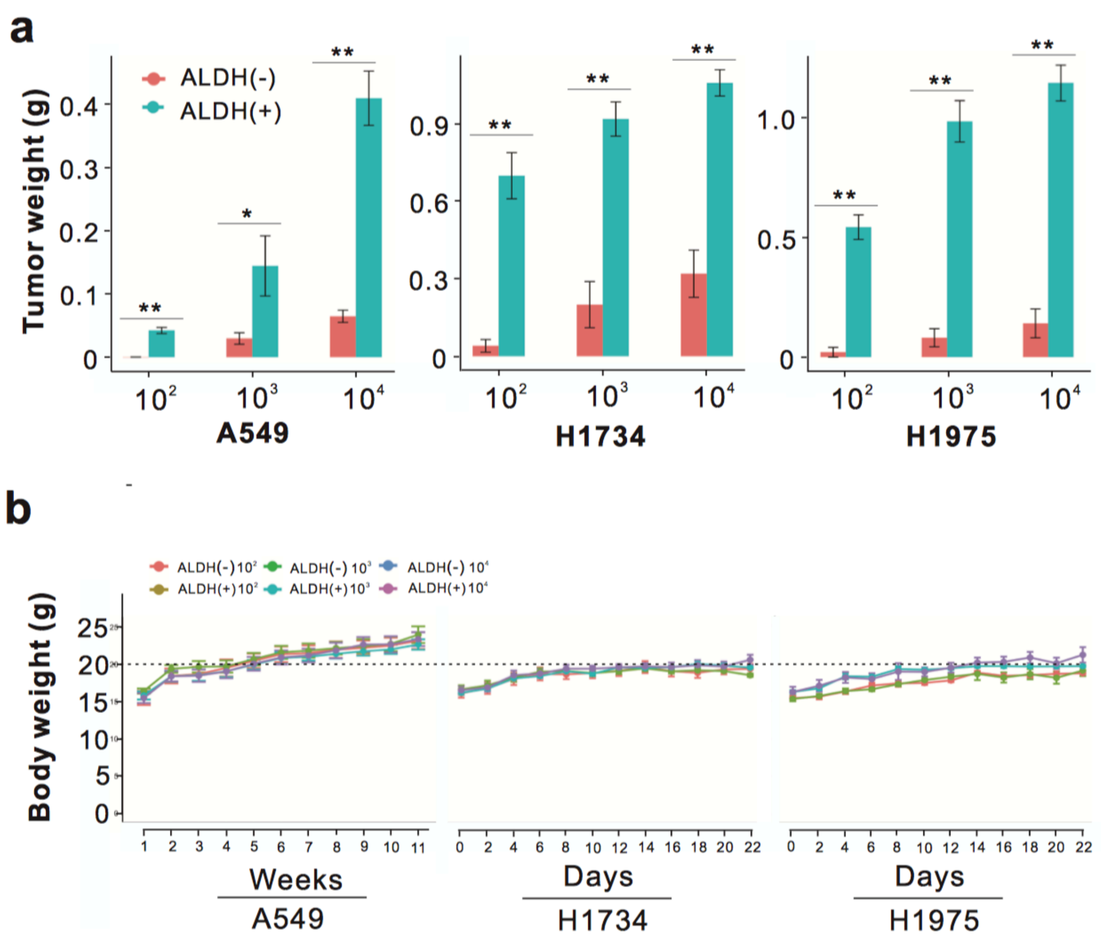

Supplementary Fig. 3. The tumor weight was evaluated after dissection of the solid subcutaneous tumors formed by different number of ALDEFLUOR-negative and ALDEFLUOR-positive cells. **p < 0.01, *p < 0.05. **b** The weight of mice injected with different cells changed over time. (Error bars represent ± SEM).

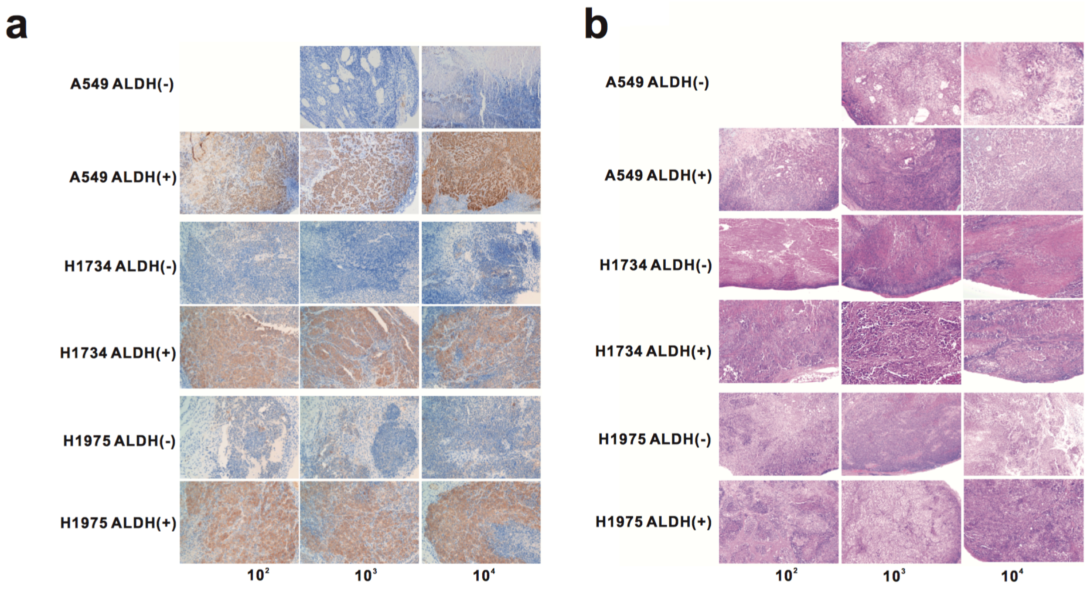

Supplementary Fig. 4. Hematoxylin and eosin staining and Immunostaining analysis. a Hematoxylin and eosin staining of xenograft tumors generated by different number of ALDEEFLUOR-positive and ALDEFLUOR-negative cells. **b** Immunostaining of xenograft tumor sections using the ALDH1 antibody.

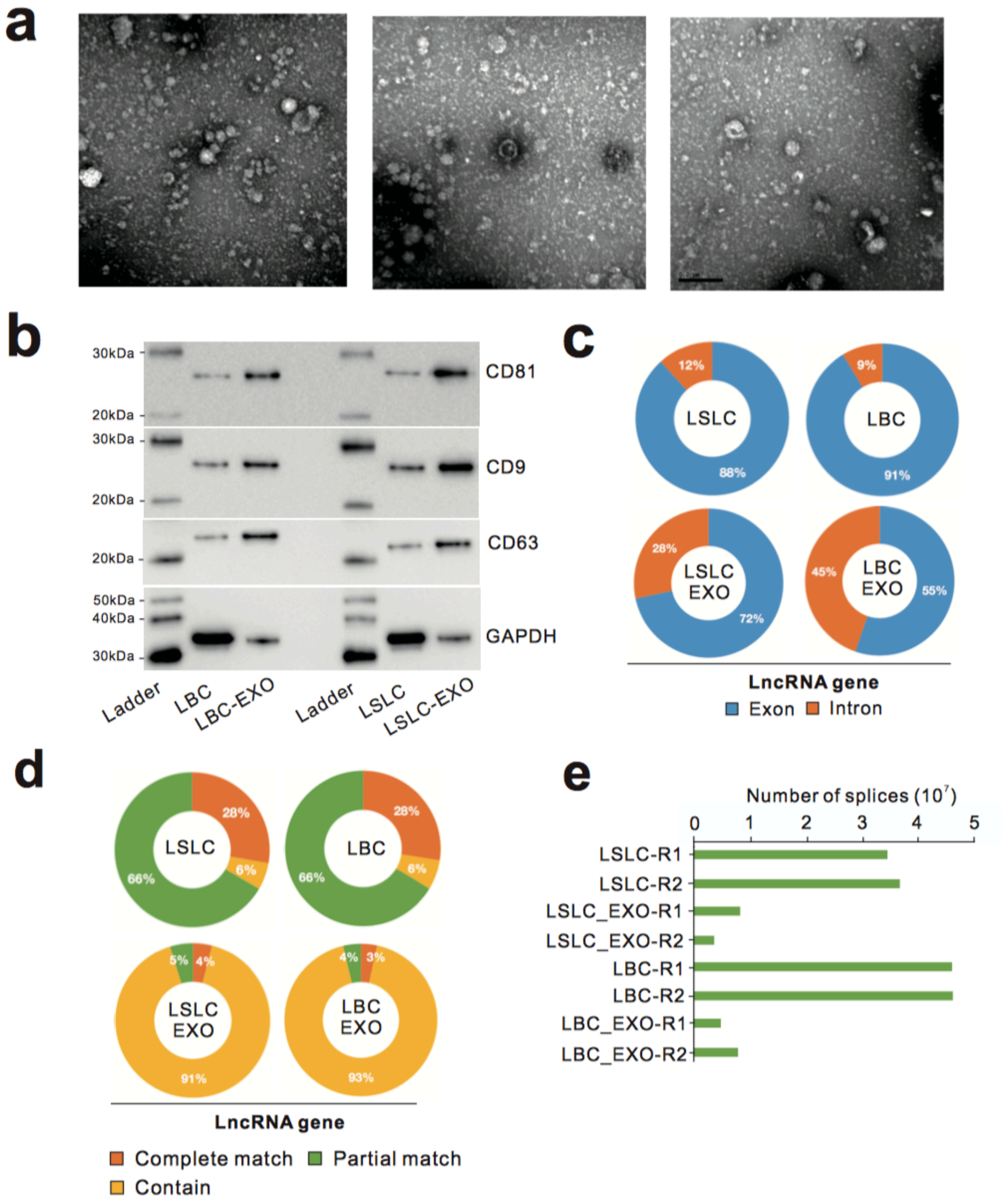

Supplementary Fig. 5. Characterization of exosomes and their compositions. a Transmission electron microscopy (TEM) imaging of exosomes isolated from three replicates of LBC. Scale bar, 200 nm. **b** The western blotting analysis of the classical exosomal markers verified the isolation of exosomes. **c** Percentage of RNA-seq reads mapping to exonic and intronic lncRNA gene regions for cellular and exosomal samples. **d** The statistics of assembled transcripts that matched to lncRNA genes. **e** The number of splice sites identified for each sample.

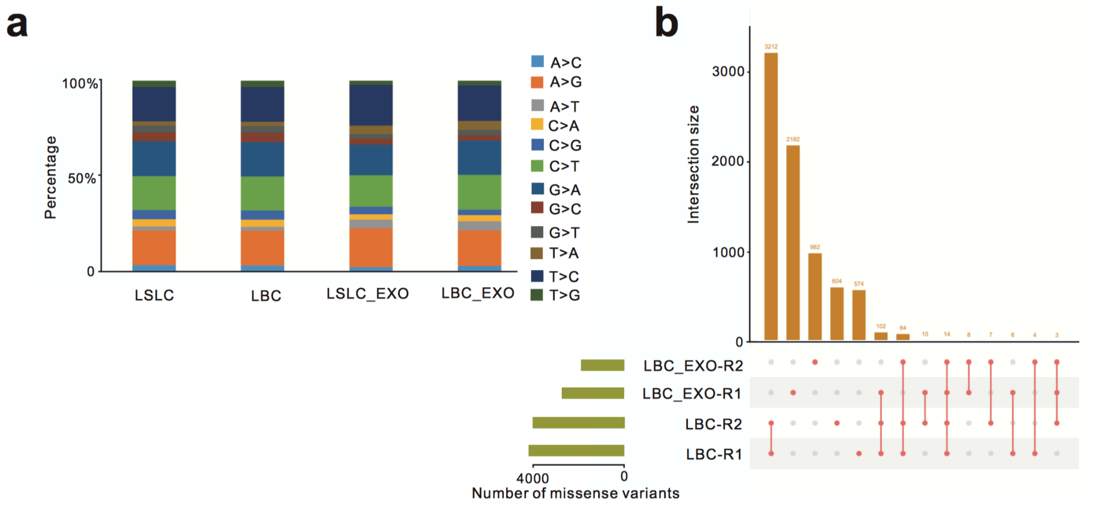

Supplementary Fig. 6. The statistics of RNA variants. a The proportion of different variant types identified from each sample type. **b** Matrix layout for all intersections of LBC and LBC-EXO samples, sorted by intersection size. Pink dots in the matrix indicate sets that are part of the intersection.

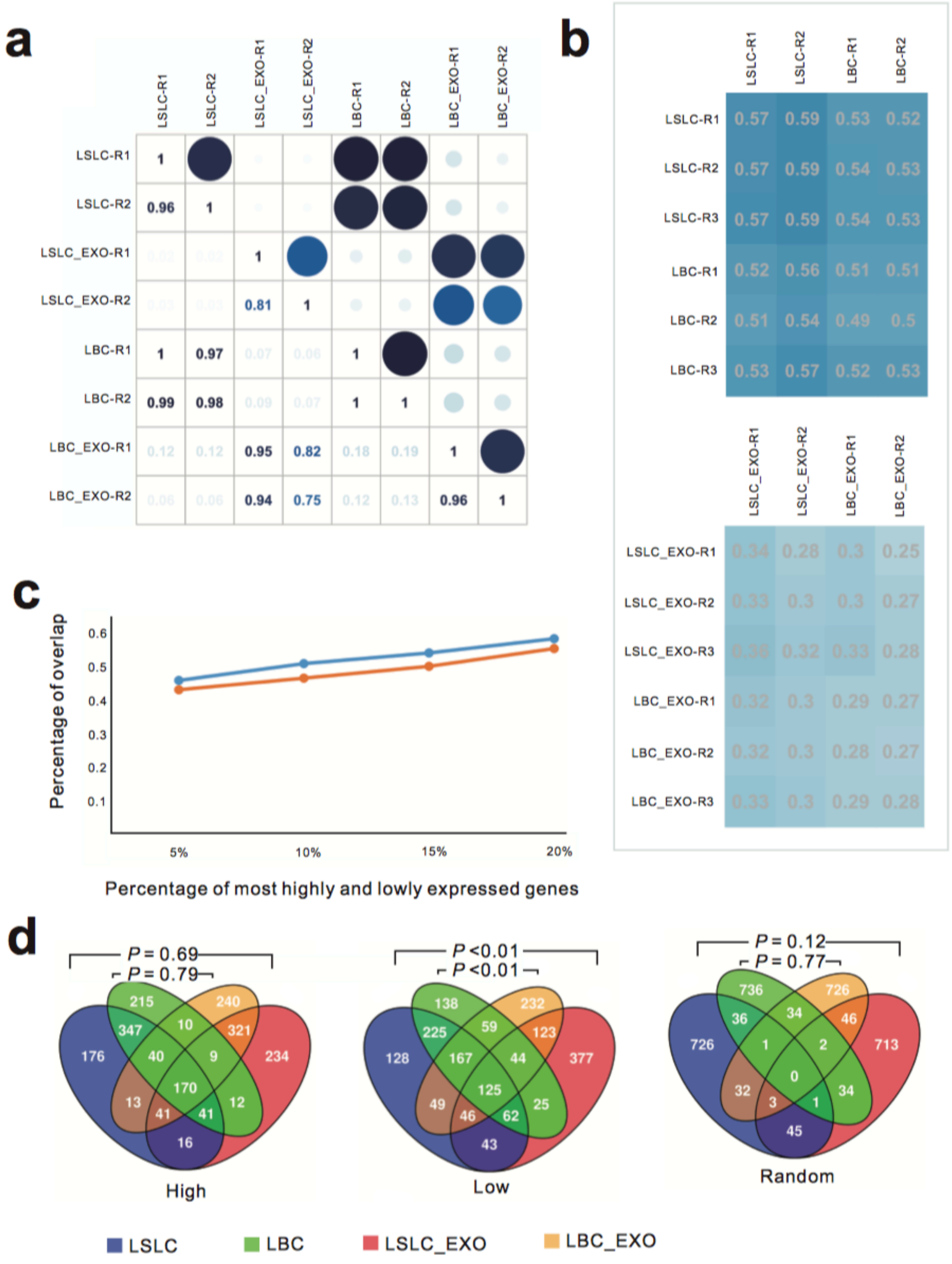

Supplementary Fig. 7. The correlation analysis of cellular and exosomal RNAs and proteins. **a** The lncRNA gene expression correlation matrix based on transcriptomic data of all samples. Color intensity and the size of the circle are proportional to the Pearson correlation coefficients. **b** The correlation matrix between protein-coding gene and protein expressions. Color intensity is proportional to the Pearson correlation coefficients. **c** The overlap statistics of the most highly (blue line) and lowly (orange line) expressed genes between LSLC and LSLC-EXO across different

cutoffs. **d** Venn diagrams depict the overlap of lncRNA genes among LSLC, LSLC-EXO, LBC and LBC-EXO samples. The *P* values were calculated by hypergeometric tests.

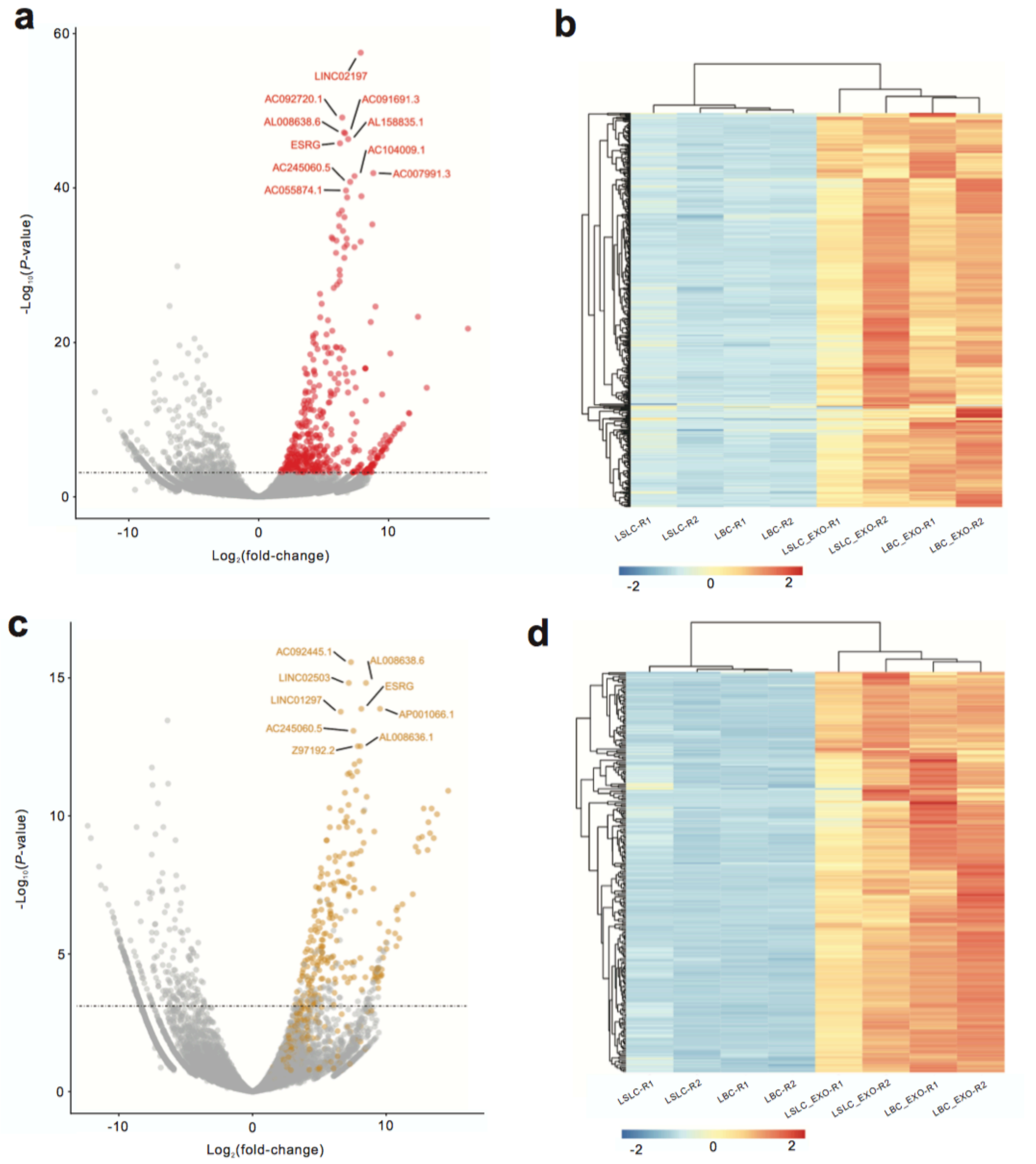

Supplementary Fig. 8. Identification and functional characterization of exosomal-enriched RNAs and proteins. **a** Volcano plots showing LSLC-EXO-enriched lncRNA genes identified using transcriptomic data. Red dots denote the genes passing our *P* value and fold difference thresholds. **b** Heatmap showing the protein-coding genes that are differentially expressed, both between LSLC and LSLC-EXO and between LBC and LBC-EXO. **c** Volcano plots showing LBC-EXO-enriched lncRNA genes. Yellow dots denote the LSLC-EXO-enriched lncRNA genes. **d** Heatmap showing the lncRNA genes that are differentially expressed, both between LSLC and LSLC-EXO and between LBC and LBC-EXO.

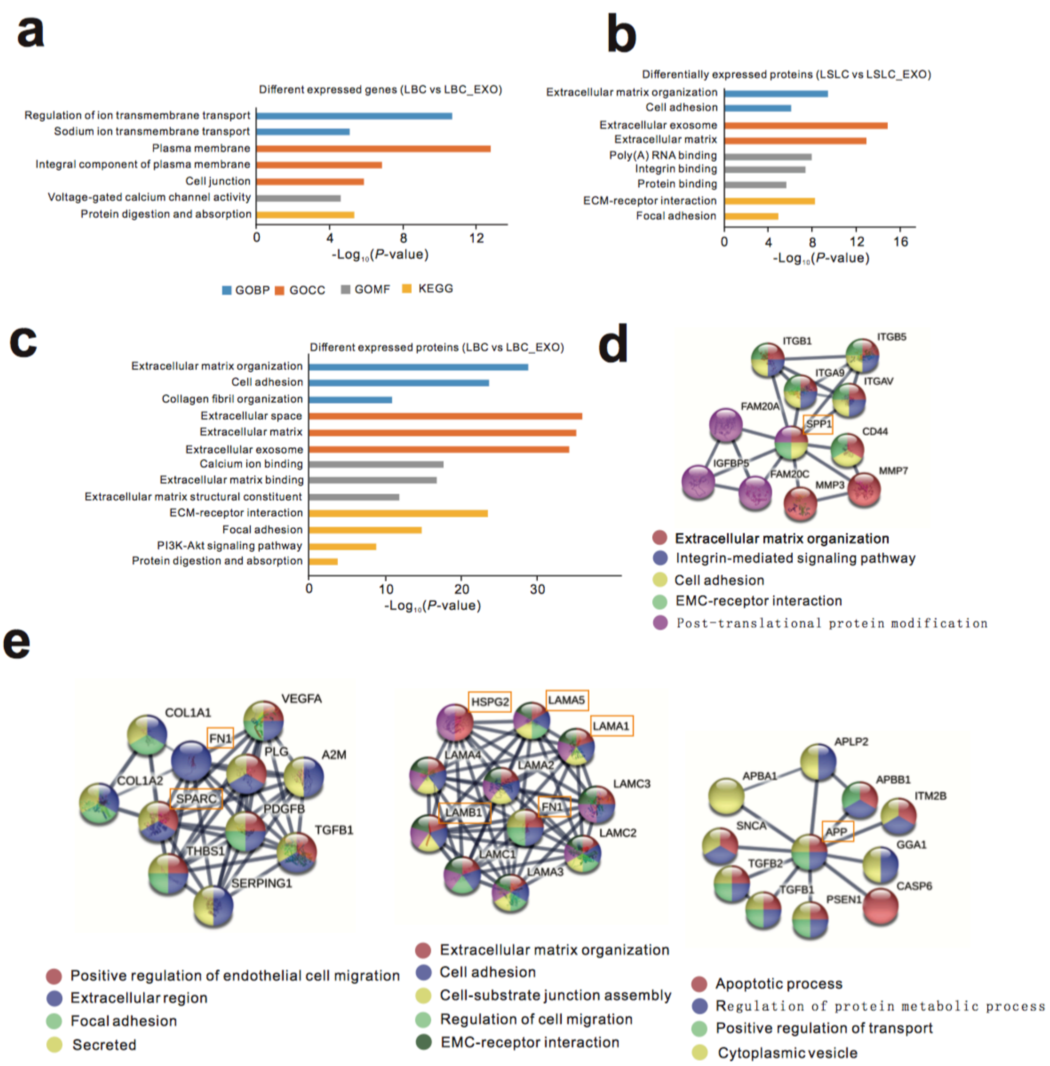

Supplementary Fig. 9. Identification and functional characterization of exosomal-enriched RNAs and proteins. **a** Functional annotation of differentially expressed proteins between LSLC and LSLC-EXO. **b** The PPI subnetwork of LSLC-EXO-enriched protein SPP1 obtained from the STRING database. **c, d** Functional annotation of differentially expressed genes (c) and proteins (d) between LBC and LBC-EXO. **e** The PPI subnetwork of representative LSLC-EXO-enriched proteins obtained from the STRING database.

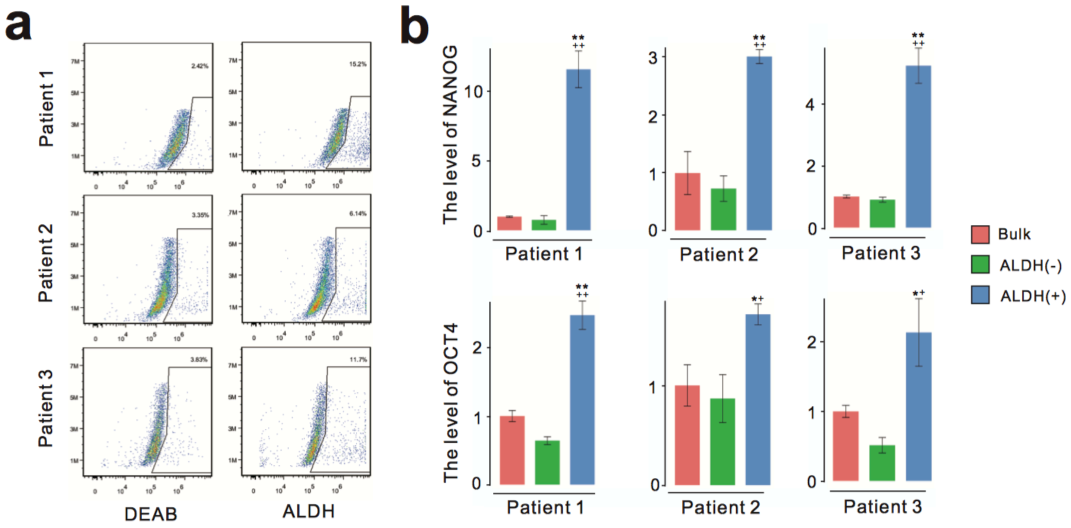

Supplementary Fig. 10. Characterization of ALDEFLUOR-positive cells from tumor tissues of lung adenocarcinoma patients. a Flow cytometry analysis of ALDH activity in tumor tissues from three patients. The specific inhibitor of DEAB and ALDH were used to establish the baseline fluorescence and to define the ALDEFLUOR-positive region, respectively. **b** The levels of stem genes (NANOG and OCT4) of bulk cells, ALDEFLUOR-negative cells, and ALDEFLUOR-positive cells from three tumor tissues were analyzed by qPCR. $^{**}p < 0.01$, $^{*}p < 0.05$, when compared with bulk cells. $^{++}p < 0.01$, $^{+}p < 0.05$, when compared with ALDEFLUOR-negative cells.

a

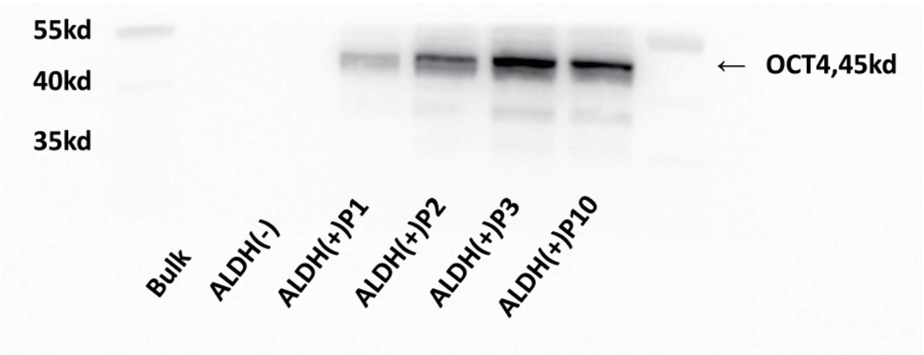

b

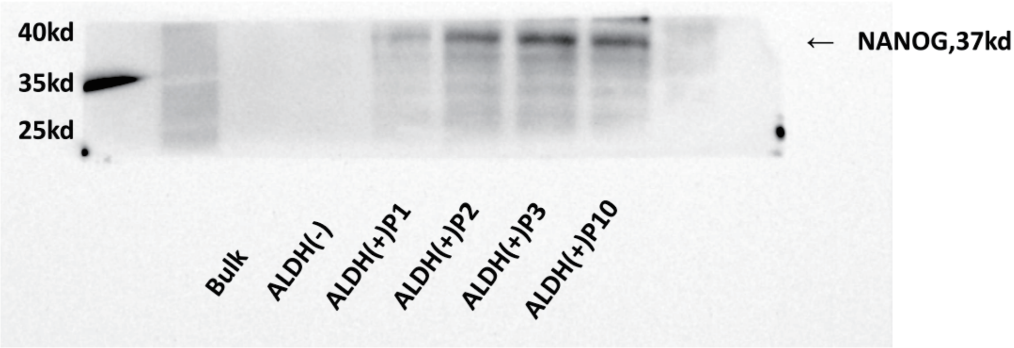

c

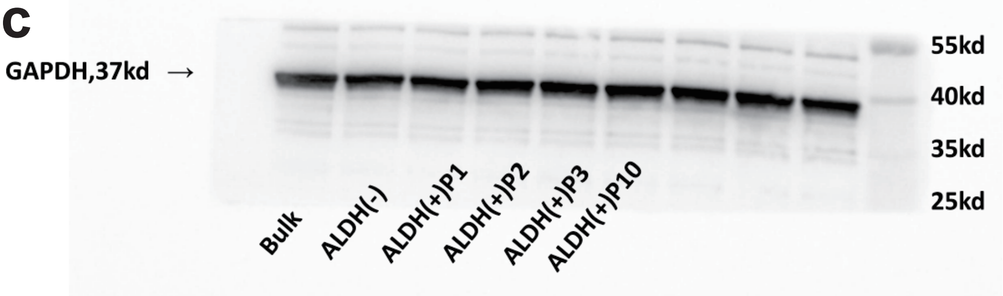

Supplementary Fig. 11. Un-cropped images of the western blots of NANOG and OCT4 in bulk cells, ALDEFLUOR-negative cells, and ALDEFLUOR-positive cells with different passage from H1975 cell lines.

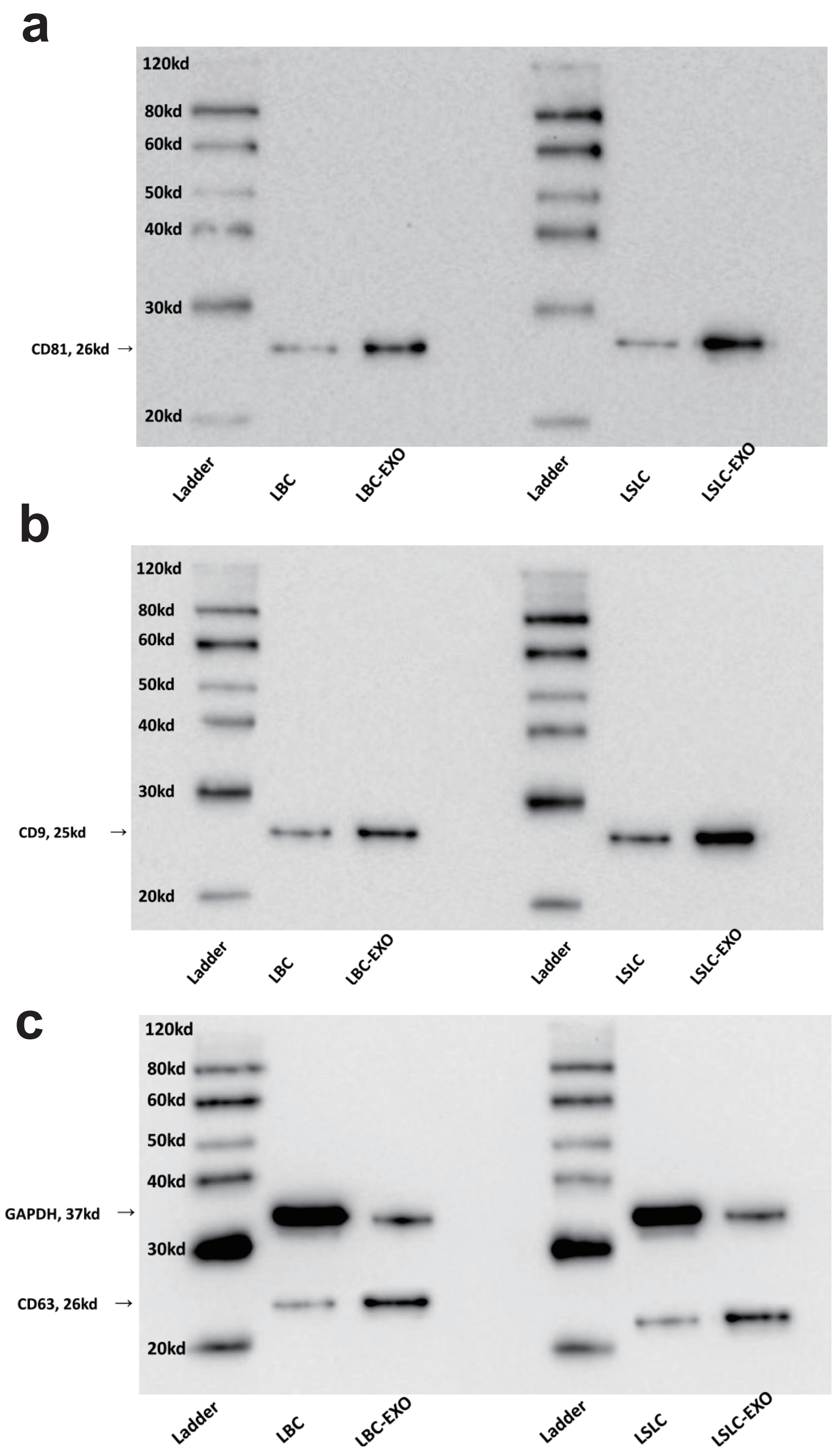

Supplementary Fig. 12. Un-cropped images of western blots of the classical exosomal markers.

Supplementary Data Sets:

Supplementary Data 1. The list of filtered RNA variants

Supplementary Data 2. The list of identified circular RNAs that matched to reference

Supplementary Data 3. The proteomic data from cells and exosomes

Supplementary Data 4. Function annotation results of common highly expressed genes and proteins

Supplementary Data 5. The lists of exosomal-enriched protein-coding and lncRNA genes

Supplementary Data 6. Function annotation results of exosomal-enriched protein-coding genes

Supplementary Data 7. The lists of exosomal-enriched proteins

Supplementary Data 8. Function annotation results of exosomal-enriched proteins

Supplementary Data 9. The lists of LSLC markers

Supplementary Data 10. Function annotation results of LSLC markers

Supplementary Data 11. The lists of genes and proteins that significantly highly expressed in both LSLC and LSLC-EXO

Supplementary Data 12. The proteomic data from patient samples

Supplementary Data 13. The list of LSLC markers validated by patient samples

Supplementary Data 14. The list of software packages used for this study
