# Supplementary material for: Dissecting the multi-omics atlas of the exosomes released by human lung adenocarcinoma stem-like cells
Source: NPJ Genom Med. 2021 Jun 14;6:48. doi: 10.1038/s41525-021-00217-5 (PMC8203745; doi:10.1038/s41525-021-00217-5)
Supplement: Supplementary file 16 — Reporting Summary [file 41525_2021_217_MOESM16_ESM.pdf]

nature research

Corresponding author(s): Fu-Rong Li,Rui-Jun Tian

Last updated by author(s): May 7, 2021

Reporting Summary

Nature Research wishes to improve the reproducibility of the work that we publish. This form provides structure for consistency and transparency in reporting. For further information on Nature Research policies, see our Editorial Policies and the Editorial Policy Checklist.

Statistics

For all statistical analyses, confirm that the following items are present in the figure legend, table legend, main text, or Methods section.

n/a	Confirmed	
☐	☒	The exact sample size (*n*) for each experimental group/condition, given as a discrete number and unit of measurement
☐	☒	A statement on whether measurements were taken from distinct samples or whether the same sample was measured repeatedly
☐	☒	The statistical test(s) used AND whether they are one- or two-sided<br>*Only common tests should be described solely by name; describe more complex techniques in the Methods section.*
☒	☐	A description of all covariates tested
☒	☐	A description of any assumptions or corrections, such as tests of normality and adjustment for multiple comparisons
☐	☒	A full description of the statistical parameters including central tendency (e.g. means) or other basic estimates (e.g. regression coefficient) AND variation (e.g. standard deviation) or associated estimates of uncertainty (e.g. confidence intervals)
☐	☒	For null hypothesis testing, the test statistic (e.g. *F*, *t*, *r*) with confidence intervals, effect sizes, degrees of freedom and *P* value noted<br>*Give P values as exact values whenever suitable.*
☒	☐	For Bayesian analysis, information on the choice of priors and Markov chain Monte Carlo settings
☒	☐	For hierarchical and complex designs, identification of the appropriate level for tests and full reporting of outcomes
☒	☐	Estimates of effect sizes (e.g. Cohen's *d*, Pearson's *r*), indicating how they were calculated

Our web collection on statistics for biologists contains articles on many of the points above.

Software and code

Policy information about availability of computer code

Data collection	The reference gene models were downloaded from the GENCODE database (version 35). The transcriptomic and proteomic data other types of exosomes were obtained from ExoRbase and ExoCarta database.
Data analysis	All of the software packages used in this study can be obtained from Supplementary Table 14.

For manuscripts utilizing custom algorithms or software that are central to the research but not yet described in published literature, software must be made available to editors and reviewers. We strongly encourage code deposition in a community repository (e.g. GitHub). See the Nature Research guidelines for submitting code & software for further information.

Data

Policy information about availability of data

All manuscripts must include a data availability statement. This statement should provide the following information, where applicable:

- Accession codes, unique identifiers, or web links for publicly available datasets
- A list of figures that have associated raw data
- A description of any restrictions on data availability

Raw RNA sequencing data has been deposited in the Sequence Read Archive (SRA) (accession number: PRJNA663998). The mass spectrometry proteomics data has been deposited to iProX database (an official member of ProteomeXchange Consortium) (iProX ID: IPX0002790000, ProteomeXchange ID: PDX023981). All processed files including identified SNVs, circRNAs, and quantified proteins are made available as supplementary tables.

Field-specific reporting

Please select the one below that is the best fit for your research. If you are not sure, read the appropriate sections before making your selection.

☒ Life sciences ☐ Behavioural & social sciences ☐ Ecological, evolutionary & environmental sciences

For a reference copy of the document with all sections, see nature.com/documents/nr-reporting-summary-flat.pdf

Life sciences study design

All studies must disclose on these points even when the disclosure is negative.

Sample size	No sample size calculations were performed. Two or three biological replicates were carried out for each experiment.
Data exclusions	N/A
Replication	Replication was successful.
Randomization	Not applicable for this study.
Blinding	Not applicable for this study.

Reporting for specific materials, systems and methods

We require information from authors about some types of materials, experimental systems and methods used in many studies. Here, indicate whether each material, system or method listed is relevant to your study. If you are not sure if a list item applies to your research, read the appropriate section before selecting a response.

Materials & experimental systems

n/a	Involved in the study	
☐	☒	Antibodies
☐	☒	Eukaryotic cell lines
☒	☐	Palaeontology and archaeology
☐	☒	Animals and other organisms
☐	☒	Human research participants
☒	☐	Clinical data
☒	☐	Dual use research of concern

Methods

n/a	Involved in the study	
☒	☐	ChIP-seq
☐	☒	Flow cytometry
☒	☐	MRI-based neuroimaging

Antibodies

Antibodies used	APC mouse IgG1, κIsotype Control (5μL/test, 5400120, Biolegend, CA, USA), FITC mouse IgG1, κIsotype control (5μL/test, 400108, Biolegend, CA, USA), anti-human CD24-FITC (5μL/test, 311104, Biolegend, CA, USA), and anti-human CD44-APC (5μL/test, 338806, Biolegend, CA, USA). CD63 (1:1000, ab134045, Abcam, Cambridge, UK), CD9 (1:2000, ab92726, Abcam, Cambridge, UK), CD81 (1:1000, ab109201, Abcam, Cambridge, UK), OCT4 (1:1000, ab19857, Abcam, Cambridge, UK), and NANOG (1:1000, ab109250, Abcam, Cambridge, UK).
Validation	For validation, the isotypes were introduced as negative controls to test the specificity of primary antibodies.

Eukaryotic cell lines

Policy information about cell lines

Cell line source(s)	The human NSCLC cell lines A549, H1734 and H1975 were obtained from the American Type Culture Collection.
Authentication	N/A
Mycoplasma contamination	Cells were tested negative for mycoplasma.
Commonly misidentified lines (See ICLAC register)	N/A

Animals and other organisms

Policy information about studies involving animals; ARRIVE guidelines recommended for reporting animal research

Laboratory animals	4-7 week-old female Balb/c nude mice.
Wild animals	N/A
Field-collected samples	N/A
Ethics oversight	This study was approved by the Institutional Ethics Committees at Shenzhen People's Hospital (Shenzhen, China).

Note that full information on the approval of the study protocol must also be provided in the manuscript.

Human research participants

Policy information about studies involving human research participants

Population characteristics	Characteristics of NSCLC patients which freshly resected tumors were collected: P1, female, 55, pathologically diagnosed with NSCLC;P2, female, 60, pathologically diagnosed with NSCLC; P3,female, 61, pathologically diagnosed with NSCLC.
Recruitment	Three patients who were pathologically diagnosed with NSCLC were enrolled in this study.
Ethics oversight	This study was approved by the Institutional Ethics Committees at Shenzhen People's Hospital (Shenzhen, China).

Note that full information on the approval of the study protocol must also be provided in the manuscript.

Flow Cytometry

Plots

Confirm that:

- [x] The axis labels state the marker and fluorochrome used (e.g. CD4-FITC).
- [x] The axis scales are clearly visible. Include numbers along axes only for bottom left plot of group (a 'group' is an analysis of identical markers).
- [x] All plots are contour plots with outliers or pseudocolor plots.
- [x] A numerical value for number of cells or percentage (with statistics) is provided.

Methodology

Sample preparation	The following monoclonal antibodies were used for staining: APC mouse IgG1, κIsotype Control (5µL/test, 5400120, Biolegend, CA, USA), FITC mouse IgG1, κIsotype control (5µL/test, 400108, Biolegend, CA, USA), anti-human CD24-FITC (5µL/test, 311104, Biolegend, CA, USA), and anti-human CD44-APC (5µL/test, 338806, Biolegend, CA, USA). Single-cell suspension was resuspended in 300 µL PBS with 3% FBS and stained with monoclonal antibodies for 15 min in the dark at room temperature. Then, cells were resuspended with PBS containing 3% FBS and analyzed using a flow cytometer.
Instrument	Beckman DxFLEX
Software	FlowJo_V10
Cell population abundance	Isotypes were introduced as negative control to eliminate the non-specific staining.
Gating strategy	Gating was based on antibodies and isotypes (1 ug for 1 million cells)that were added to the cells.

- [x] Tick this box to confirm that a figure exemplifying the gating strategy is provided in the Supplementary Information.
